# Eco‐friendly Regioselective Synthesis, Biological Evaluation of Some New 5‐acylfunctionalized 2‐(*1H*‐pyrazol‐1‐yl)thiazoles as Potential Antimicrobial and Anthelmintic Agents

**DOI:** 10.1002/open.202400142

**Published:** 2024-08-08

**Authors:** Ranjana Aggarwal, Manisha Sharma, Mona Hooda, Prabodh C. Sharma, Diksha Sharma

**Affiliations:** ^1^ Department of Chemistry Kurukshetra University Kurukshetra 136119 Haryana India; ^2^ Council of Scientific and Industrial Research-National Institute of Science Communication and Policy Research New Delhi 110012 India; ^3^ Department of Chemistry Gurugram University Gurugram 122003 Haryana India; ^4^ School of Pharmaceutical Science Delhi Pharmaceutical Science and Research University New Delhi 110017 India; ^5^ Swami Devi Dyal Institute of Pharmacy Golpura, Barwala 134118 India

**Keywords:** Solvent-free, 2-(*1H*-pyrazol-1-yl)thiazoles, multinuclear 2D NMR, antimicrobial, anthelmintic, structure activity relationship

## Abstract

The present study describes an eco‐friendly NBS‐assisted regioselective synthesis of new 5‐acylfunctionalized 5‐acylfunctionalized 2‐(*1H*‐pyrazol‐1‐yl)thiazoles by condensation of 3,5‐dimethyl‐1*H*‐pyrazole‐1‐carbothioamide with unsymmetrical 1,3‐diketones under solvent‐free conditions. The structural elucidation of the newly synthesized compounds was accomplished using various spectroscopic techniques viz. FTIR, NMR and mass spectrometry. All the newly synthesized compounds were examined for their *in vitro* antimicrobial potential against both pathogenic gram positive and gram negative bacterial and fungal species as well as anthelmintic activity against *Pheretima posthuma* earthworms. The results of antimicrobial activity revealed that all tested compounds **3 a–j** showed excellent antimicrobial potential particularly against *S. aureus*. It was also observed that compounds **3 e** and **3 i** (MIC=62.5 μg/mL**)** showed greater potency against *E. coli*, whereas compounds **3 a** and **3 h (**MIC=50 μg/mL and 62.5 μg/mL) demonstrated better activity against *P. aeruginosa* and compound **3 i** (MIC=62.5 μg/mL) exhibited superior activity against *S. pyogenus* when compared to standard drug Ampicillin (MIC=100μg/mL). Compound **3 e** and **3 j** revealed remarkable antifungal and anthelmintic activities. To find out binding interactions of target compounds with target proteins and pharmacokinetic parameters of the compounds, *in silico* investigations involving molecular docking studies and ADMET predictions were also performed.

## Graphical abstract

This article presents the green regioselective synthesis of novel 5‐acylfunctionalized 2‐(1*H*‐pyrazol‐1‐yl)thiazoles **3 a–j** and their biological potential against pathogenic microbes and earthworms. Compounds **3 a–j** were found to display significant to excellent activity against tested pathogens most prominently against *S. aureus. In silico* methods were used to investigate the potency of compounds against target proteins and to examine their safety *via* ADMET prediction. Compounds exhibited good *in silico* docking and ADMET profile.

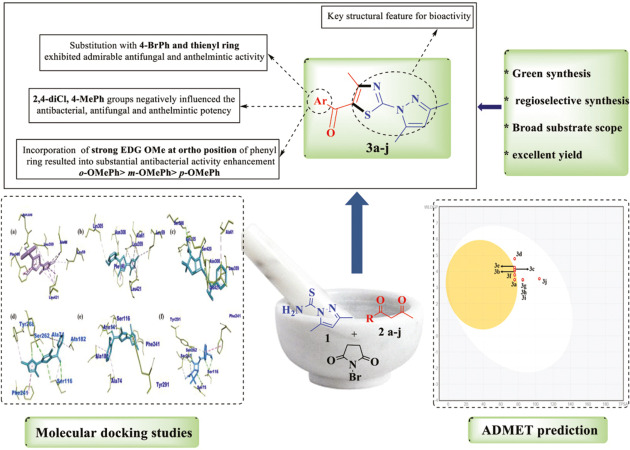

## 1 Introduction

All facets of human life are intimately impacted by microorganisms, which can be beneficial or harmful. A fundamentally new level of healthcare has been achieved by the advent of antibiotics into medical practice. As a consequence, the mortality rate from infectious diseases has declined significantly.^[1]^ Unluckily, microbial resistance to the majority of antibiotic classes has been an emerging challenge ever since.^[2]^ According to the World Health Organization (WHO), current situation with multiple drug resistance poses a severe threat to human society and continues to worsen every year.^[3]^ In the present time, nosocomial infections most commonly caused by bacteria *E. coli*, *S*. aureus, and *P. aeruginosa*, are becoming a huge burden on the healthcare system globally.^[4]^ The increasing resistance of these bacteria to clinically available antibiotics is making the situation more serious and complex. It is believed that humanity is about to enter a post‐antibiotic era, when even common infections or minor injuries can be life‐threatening.^[5]^ In this scenario, one of the most important responsibilities of contemporary medicinal chemistry is to find new antimicrobial agents with diverse pharmacophores that would constrain pathogen growth and become effective antibacterial and antifungal drugs.

**Figure 1:**
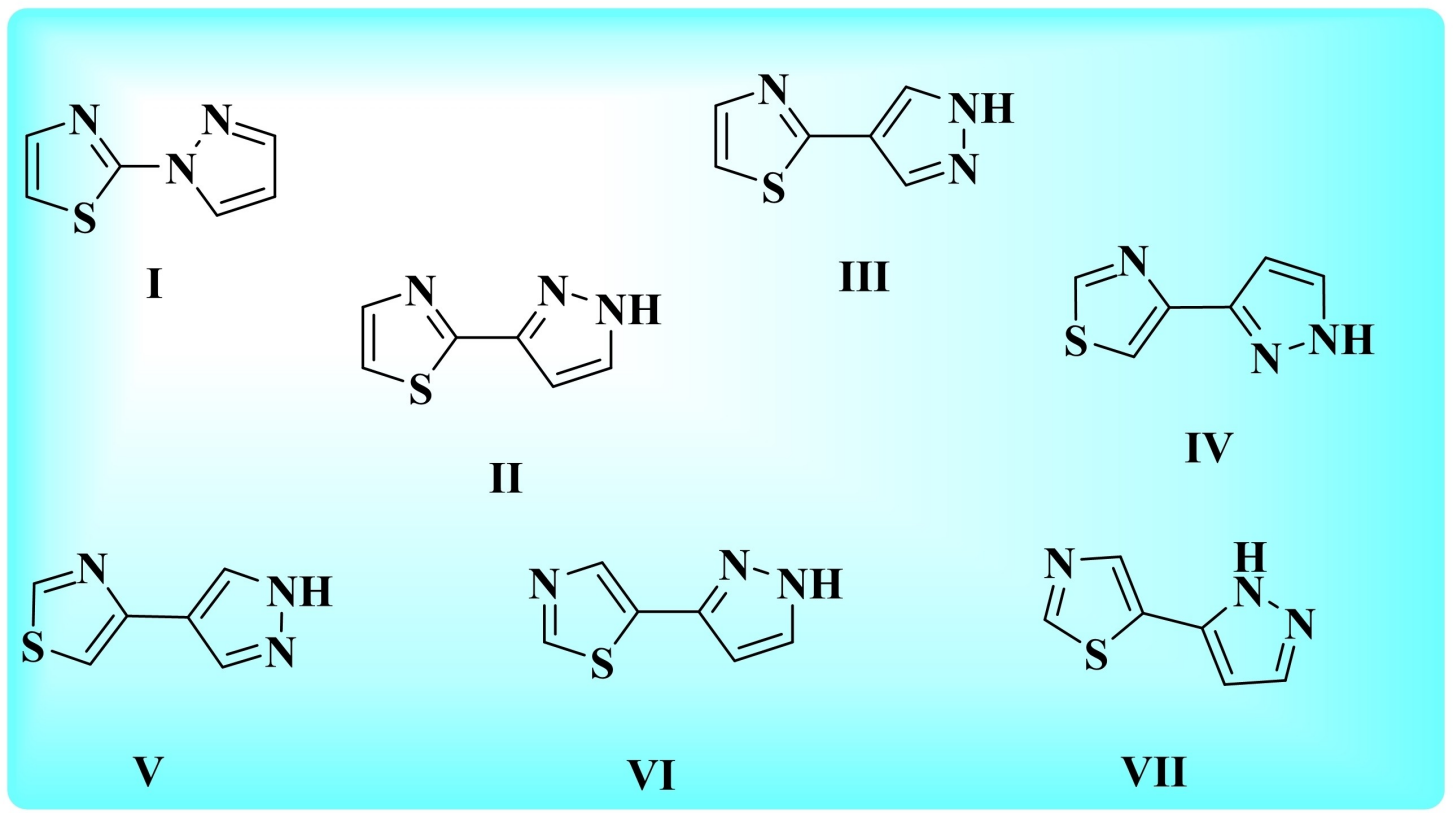
Various isomeric structural variants of pyrazolyl‐thiazole.

The pyrazole skeleton is one of the most pivotal nitrogen heterocycles of medicinal importance, supplemented with almost all the pharmacological and biological activities.^[6, 7]^ Likewise, the valuable molecular architecture of thiazoles makes them important building blocks in medicinal chemistry suitable for drug development due to their inimitable capacity to influence drug lipophilicity and therapeutic potency.^[8]^ The medicinal capability of thiazole and pyrazole derivatives can be well cited by the presence of these scaffolds in various commercial drugs.^[9, 10]^ Molecular hybridization is a frequent approach in rational drug design based on the combination of pharmacophoric moieties of different bioactive substances to produce hybrid compounds which commonly show the better activities with lower toxicity.^[11]^ Literature survey revealed that the combination of pharmacologically attractive scaffolds, pyrazole and thiazole for the creation of lead molecules have received much attention, as their grouping enhances the existing pharmacological activity many fold or imparts some additional activity.^[12, 13]^ Pyazole and thiazole may be linked in various combinations based on the substitution position of pyrazole on thiazole. On the basis of various fusion permutations of pyrazole and thiazole, there is the possibility of formation of seven isomeric structural variants of pyrazolyl‐thiazole; 2‐(*1H*‐pyrazol‐1‐yl)thiazole **I**,^[14]^ 2‐(*1H*‐pyrazol‐3‐yl)thiazole **II**,^[15]^ 2‐(*1H*‐pyrazol‐4‐yl)thiazole **III**,^[16]^ 4‐(*1H*‐pyrazol‐3‐yl)thiazole **IV**,^[17]^ 4‐(*1H*‐pyrazol‐4‐yl)thiazole **V**,^[18]^ 5‐(*1H*‐pyrazol‐3‐yl)thiazole **VI**,^[19]^ 5‐(*1H*‐pyrazol‐5‐yl)thiazole **VII** ^[20]^ (Figure 1).

**Figure 2:**
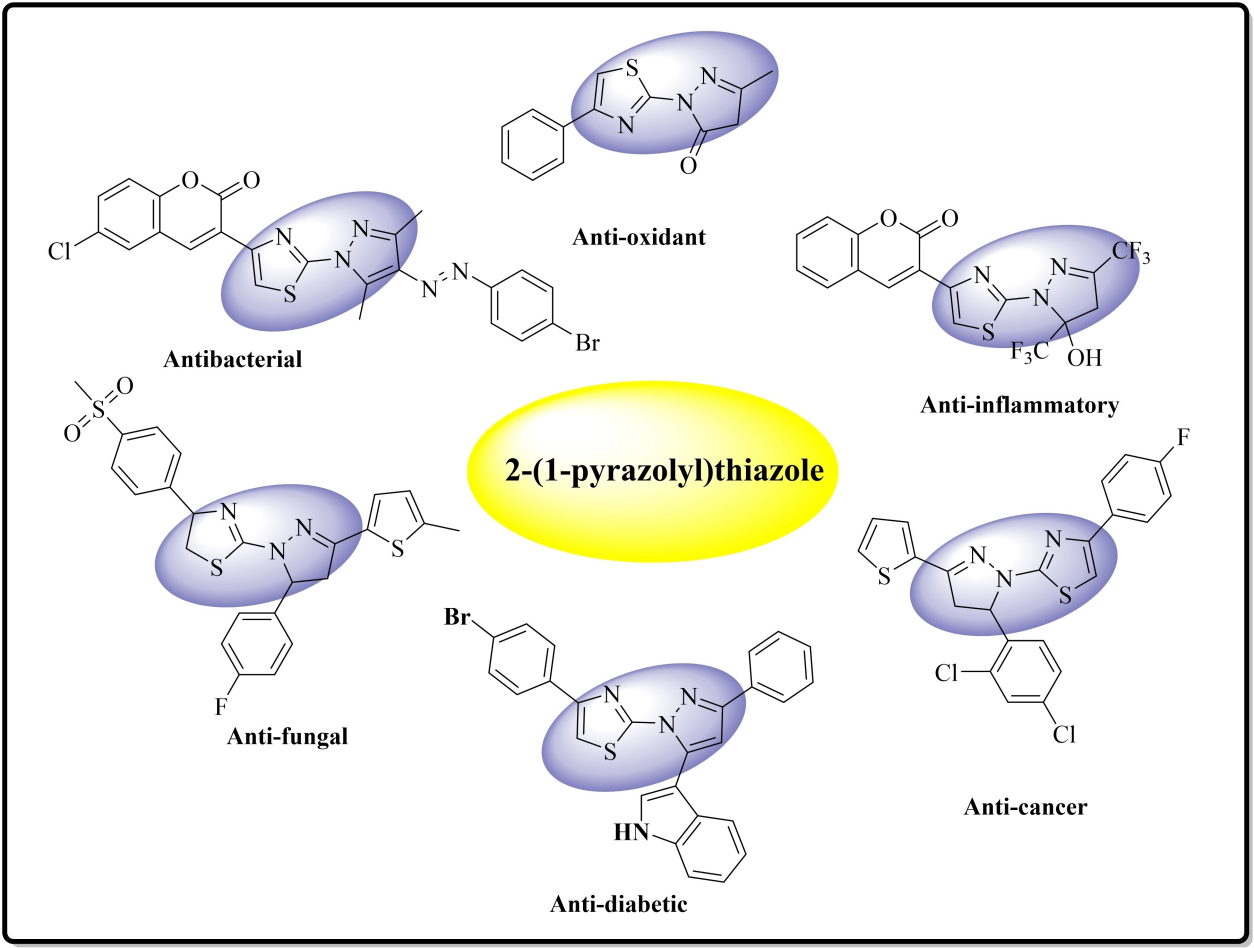
Several pharmaceutically important 2‐(*1H*‐pyrazol‐1‐yl)thiazole skeletons.

The structural diversity and associated biological profile of 2‐(*1H*‐pyrazol‐1‐yl)thiazole **I** have made this scaffold as extensively researched and sought‐after heterocyclic hybrid in recent years. 2‐(*1H*‐pyrazol‐1‐yl)thiazoles have fascinated continuing interest because of their varied therapeutic and pharmacological activities, found application in drug development for the treatment of bacterial, fungal, malarial, inflammation, HIV infections and more.[^21^, ^22^, ^23^, ^24^] Additionally, 2‐(*1H*‐pyrazol‐1‐yl)thiazole derivatives have high anticancer antidiabetic, antialzheimer, antimicrobial and antioxidant activity.[^25^, ^26^, ^27^, ^28^] Apart from the pharmacological properties, 2‐(*1H*‐pyrazol‐1‐yl)thiazoles also find applications in dyes due to their unique optical and photophysical properties.[^29^, ^30^, ^31^] Some of the representative bioactive 2‐(*1H*‐pyrazol‐1‐yl)thiazoles structures are revealed in Figure 2. Thus the search for new antibacterial and antifungal agents among pyrazole thiazole hybrid related conjugates is a promising direction in current medicinal chemistry. Further, the structural diversity of 2‐(*1H*‐pyrazol‐1‐yl)thiazoles have also made them attractive target for design and development of new antimicrobial agents.^[32]^

In view of the prevalence of 2‐(*1H*‐pyrazol‐1yl)thiazoles framework in medicinal chemistry, several synthetic methods have been developed for the synthesis of these heterocyclic compounds. Although the reported protocols show variation in terms of synthons and reaction conditions, they have various associated drawbacks such as lower regioselectivity, use of volatile and flammable organic solvents, prolonged reaction time, tedious work‐up procedures, multi‐step reactions.^[33, 34]^ So, it is desirable and challenging to develop an efficient and green protocol for the synthesis of multi functionalized 2‐(*1H*‐pyrazol‐1‐yl)thiazoles.

**Figure 3:**
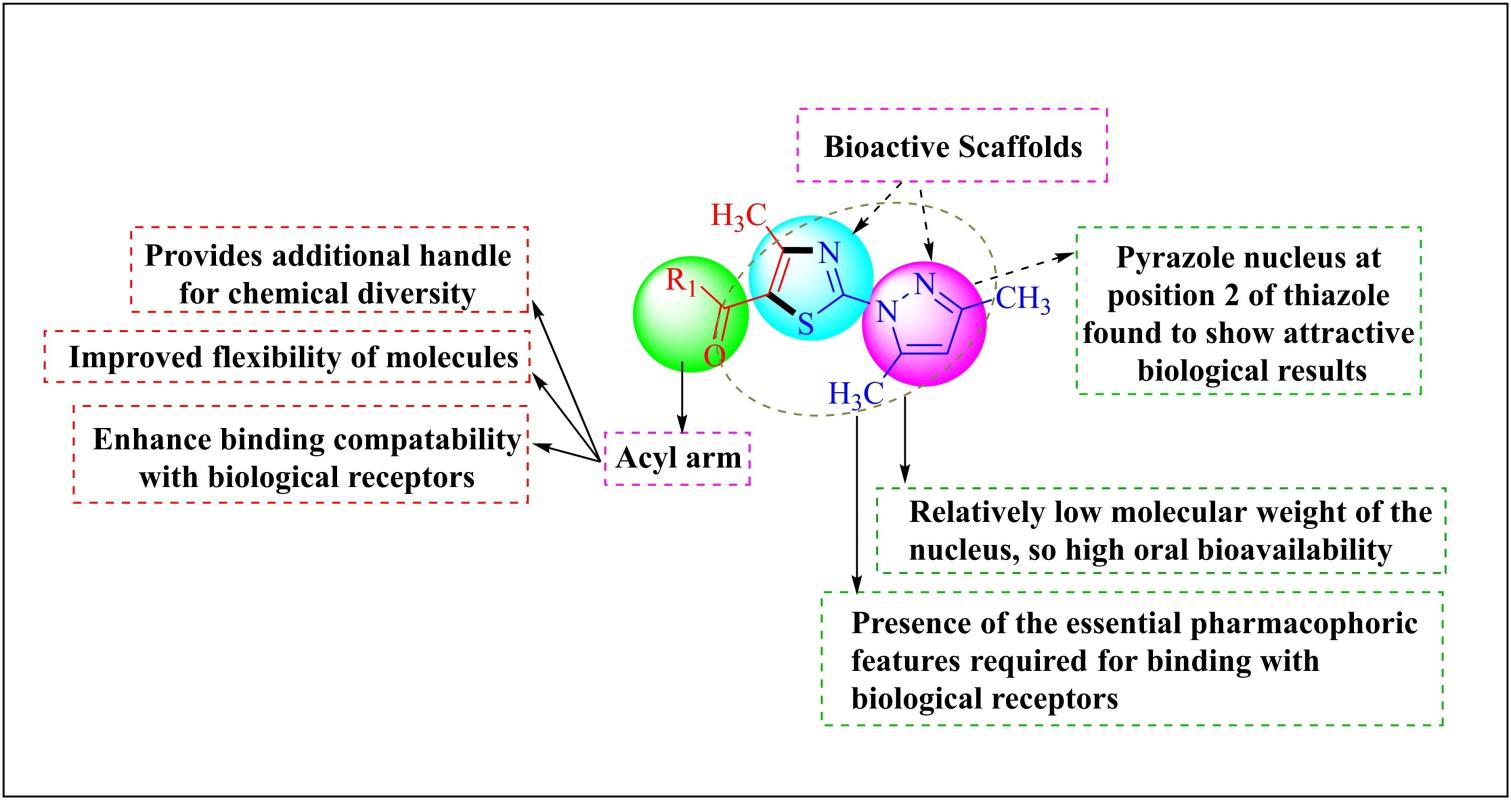
Rationale for the design of the new 5‐acyl functionalized 2‐(*1H*‐pyrazol‐1‐yl)thiazoles.

Our research team has long been interested in expending the reactivity of unsymmetrical α‐bromo‐1,3‐diketons with various bi or tri nucleophiles to synthesize different acylated azaheterocycles.[^35^, ^36^, ^37^, ^38^] Thus, from viewpoint of the promising antimicrobial activity of 2‐(*1H*‐pyrazol‐1‐yl)thiazoles and in continuation to our efforts concerning the eco‐friendly regioselective synthesis of bioactive heterocyclic compounds,[^39^, ^40^, ^41^] herein, we intended to design and synthesize 5‐acylfunctionalized 2‐(*1H*‐pyrazol‐1‐yl)thiazoles **(**Figure 3 **)**.

Therefore, in the present work, we have synthesized diversely substituted targeted compounds by regioselective reaction of unsymmetrical α‐bromo‐1,3‐diketones (generated *in situ* from the reaction of 1,3‐diketones **2** with NBS) with 3,5‐dimethyl‐1*H*‐pyrazole‐1‐carbothioamide **1** and screened for their *in vitro* antimicrobial and anthelmintic activities. To estimate the binding interaction of active compounds within active sites of enzymes and pharmacokinetic parameters of the designed derivatives, *in silico* investigations involving molecular docking studies and ADMET predictions were carried out.

## 2 Results and Discussion

### 2.1 Chemistry

The key precursors required in this study; 3,5‐dimethyl‐1*H*‐pyrazole‐1‐carbothioamide **1** was synthesized from the reaction of acetylacetone with thiosemicarbazide,^[42]^ whereas unsymmetrical 1,3‐diketones **2** were prepared as per the literature report by conventional Claisen condensation reaction of enolizable ketones with ethylacetate.^[43]^

**Scheme 1:**
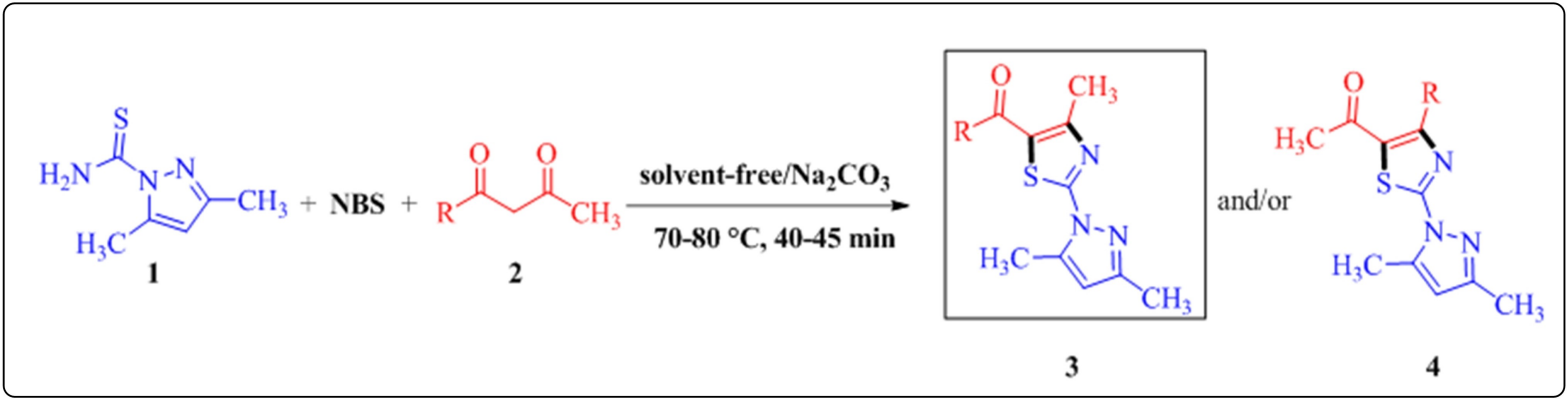
Possible regioisomers formed by reaction of **1** and **2**.

Recently, our research group have achieved the regioselective synthesis of 2‐aryl/hetaryl‐4‐methyl‐5‐acylthiazoles from the reaction of α‐bromo‐1,3‐diketones with various thioamides under solvent‐free conditions,^[35]^ interestingly, when we explored the same reaction under visible‐light irradiation, an unexpected synthesis of 3,5‐diaryl‐1,2,4‐thiadiazoles was achieved *via* oxidative dimerization of thiobenzamides.^[44]^ Taking a lead from this, we have decided to extend our research work towards regioselective synthesis of 5‐acylfunctionalized 2‐(*1H*‐pyrazol‐1‐yl)thiazoles by condensing differently substituted α‐bromo‐1,3‐diketones generated *in situ* by reaction of respective 1,3‐diketones and *N‐*bromosuccinimide with 3,5‐dimethyl‐1*H*‐pyrazole‐1‐carbothioamide under solvent‐free conditions. The precise reactivity of unsymmetrical 1,3‐dicarbonyl **2** compounds for the selective construction of functionalized organic molecules of substantial synthetic and biological potential makes them appealing synthons to prepare 5‐acylfunctionalized 2‐(*1H*‐pyrazol‐1‐yl)thiazoles.^[45]^ Theoretically, the reaction of unsymmetrical α‐bromo‐1,3‐diketones bearing two different electrophilic carbonyl centres, with binucleophilic 3,5‐dimethyl‐1*H*‐pyrazole‐1‐carbothioamide **1** provides an opportunity to produce two regioisomeric products (Scheme 1).

For the synthesis of targeted 5‐acylfunctionalized 2‐(*1H*‐pyrazol‐1‐yl)thiazoles under environmentally benign solvent‐free conditions, initially **2 a** was ground with *NBS* in equimolar amounts until TLC confirmed complete consumption of **2 a**. Subsequently, 3,5‐dimethyl‐1*H*‐pyrazole‐1‐carbothioamide **2** was added in equimolar amount. The resulting mixture ground proficiently in presence of base; Na_2_CO_3_ (1.2 eq.) at room temperature for 30 min. The reaction was monitored by TLC (ethyl acetate: petroleum ether, 20 : 80, *v/v* as eluent) at regular intervals. TLC indicated the partial consumption of reactants. Then, the reaction mixture transferred to a conical flask and heated at 70–80 °C. TLC specified the completion of the reaction in just 40 min, and then the reaction mixture was cooled to room temperature, diluted with water and extracted with ethylacetate (2×25 mL). The combined organic extract was dried over Na_2_SO_4_ and concentrated which resulted into the formation of exclusively single product, out of two possible regioisomeric products with 85 % yield **(**Scheme 1 **)**.

Appearance of only one IR characteristic absorption band at 1628 cm^−1^corresponding to the carbonyl group indicating the formation of **3 a**. In ^1^H NMR spectrum, presence of three singlets of three proton intensity at δ 2.26, 2.62 and 2.69 ppm in aliphatic region, a sharp singlet at δ 6.02 ppm integrating for one proton correspond to pyrazole 4‐H and peculiar pattern of phenyl protons in aromatic region 7.45–7.83 ppm confirm the formation of **3 a**. ^13^C NMR spectrum of compound **3 a** showed three signals at δ 18.8, 13.9, 13.6 due to three methyl carbons and ten signals in chemical shift range of 110.4–163.7 ppm due to thiazole and pyrazole carbons and a signal at 188.5 ppm due to carbonyl carbon respectively, which clearly indicated the successful condensation of reactants into product **3 a**. The HRMS (ESI) m/z calculated for C_16_H_15_N_3_OS is 297.0936 and was observed at 298.0914 for (M+H)^+^ also confirmed the product formation.

After that, the conclusive evidence for the formation of regioisoer (2‐(3,5‐dimethyl‐1H‐pyrazol‐1‐yl)‐4‐methylthiazol‐5‐yl)(phenyl)methanone **3 a** and chemical shift values for methyl substituents in **3 a** were obtained by performing multinuclear 2D NMR experiments; (^1^H−^13^C) HMBC, (^1^H−^13^C) HSQC, (^1^H−^15^N) HMBC.

**Figure 4:**
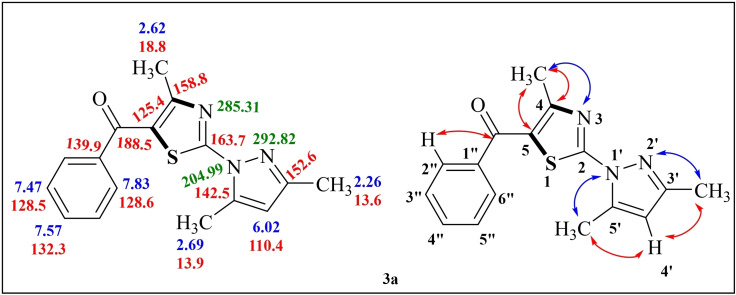
NMR chemical shift values and 2D NMR correlations diagram for compounds **3 a**.

The (^1^H−^13^C) HMBC spectrum revealed that carbonyl carbon at δ 188.5 ppm showed a cross peak with H2“/H6” (δ 7.83) protons of aryl ring confirming the presence of CO with aryl/hetaryl ring which shows the product obtained as regioisomer **3 a**. (^1^H−^13^C) HSQC correlated the carbon at δ 110.4 with C_4_ proton of pyrazole ring. The (^1^H−^13^C) HMBC showed correlation of two methyl protons at δ 2.26, 2.69 with carbon at δ110.4 which clearly indicated that these two methyl protons belonged to pyrazole ring. It has been well recognized that due to a lone pair of electrons present on the nitrogen atom of the thiazole nucleus, 5‐CH_3_ is more deshielded compared to 3‐CH_3_ and therefore appeared at about δ 2.69 ppm. The residual methyl protons at δ 2.62 showed cross‐peaks with C‐4 (δ 158.8) and C‐5 (δ 125.4), thus confirming the presence of methyl substituent at position‐4 of thiazole nucleus. All the correlation exhibited by methyl protons with nitrogen in ^1^H−^15^N HMBC spectrum (Figure 4 **)** also supported the assignment of product as regioisomer **3 a**. Thus, from the above 2D NMR evidence structure can unequivocally be assigned to (2‐(3,5‐dimethyl‐1H‐pyrazol‐1‐yl)‐4‐methylthiazol‐5‐yl)(phenyl)methanone **3 a**. The 2D NMR correlation results obtained for compounds **3 a** are presented in the supplementary material (Table S1).

After assignment the structure of regioisomer, the optimized reaction conditions were then applied to diverse unsymmetrical 1,3‐diketones decorated with variety of EDG/EWG/heteroaryl functional group such as Me, OMe, F, Cl, Br, thienyl etc. As shown in Table 1, all reaction combinations underwent successful condensation to give the desired product in 79–91 % yields. However the reaction with a 1,3‐diketone containing strong electron‐withdrawing groups (such as NO_2_) was not completed even after grinding 4 h.

**Table 1:**
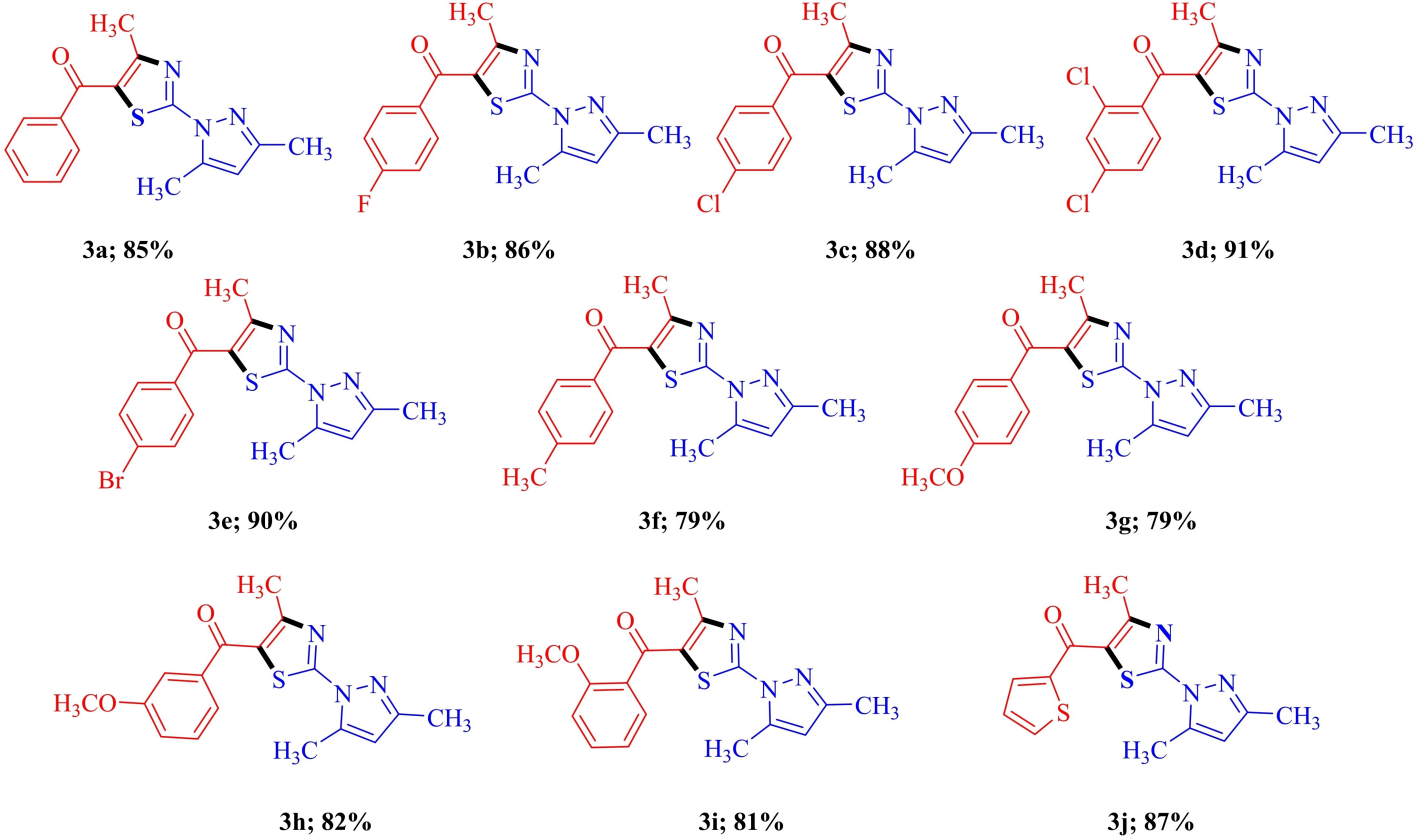
Scope of unsymmetrical 1,3‐diketones.^[a,b]^

^[a]^ Reaction conditions: 1,3‐diketones **2** (1.0 mmol), 3,5‐dimethyl‐1H‐pyrazole‐1‐carbothioamide **1** (1.0 mmol) and NBS(1.0 mmol) were ground in pestle mortar for 40–45 min;^[b]^ isolated yields.

### 2.2 Mechanism

**Scheme 2:**
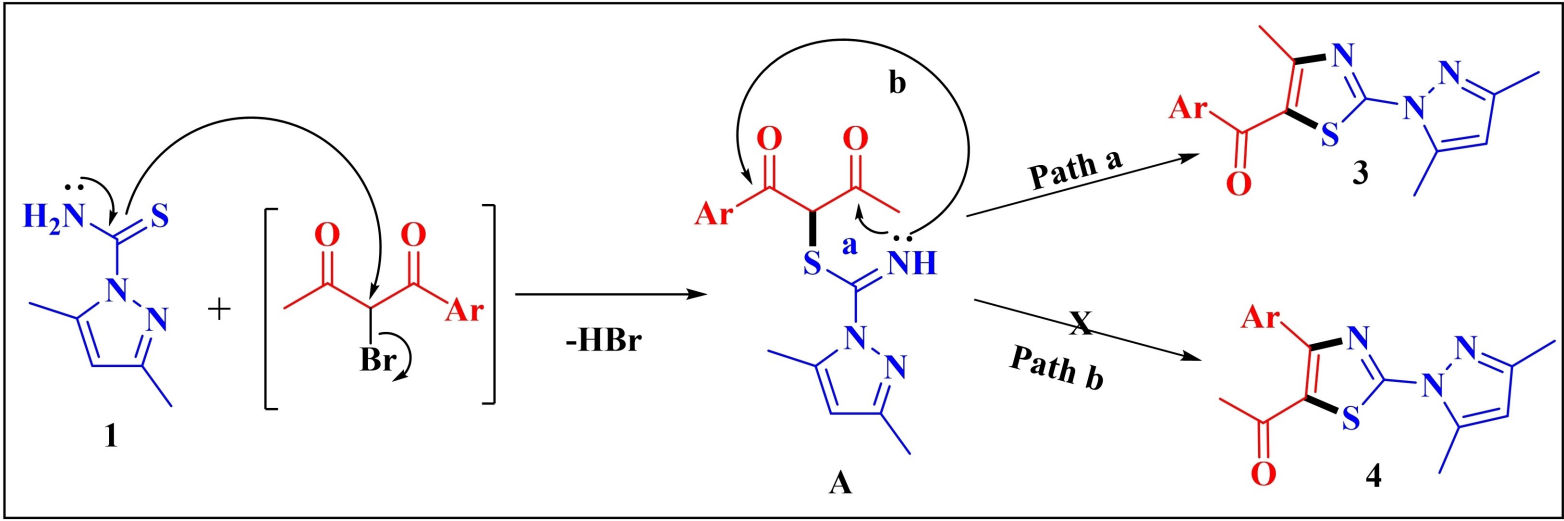
Plausible mechanism for regioselective synthesis of 5‐acylfunctionalized 2‐(*1H*‐pyrazol‐1‐yl)thiazoles **3 a**–**j**.

The plausible mechanism for regioselective synthesis of 5‐acylfunctionalized 2‐(1*H*‐pyrazol‐1‐yl)thiazoles **3** is outlined in Scheme 2. The reaction seems to be triggered by the initial displacement of bromine from α‐bromo‐1,3‐diketone by the sulphur of 3,5‐dimethyl‐1*H*‐pyrazole‐1‐carbothioamide **1** to give *S*‐alkylated open chain intermediate **A**, followed by the nucleophilic addition of imine nitrogen to the less sterically hindered carbonyl carbon (**path a)** to form 5‐acylfunctionalized 2‐(*1H*‐pyrazol‐1‐yl)thiazoles **3 a**–**j** as the final product.

### 2.3 Biological Evaluation

#### 2.3.1 Antimicrobial Activity

All the synthesized 5‐acylfunctionalized 2‐(*1H*‐pyrazol‐1‐yl)thiazoles analogs **3 a**–**j** were assayed for their *in vitro* antimicrobial potential against two Gram‐positive; *Staphylococcus. aureus*, *Streptococcus. pyogenes*as well as two Gram‐negative bacterial species; *Escherichia coli*, *Pseudomonas aeruginosa* and three fungal species; *Candida albicans*, *Aspergillus niger*, *Aspergillus clavatus*. The activity was reported by measuring the minimum inhibitory concentrations (MICs) by using broth micro dilution assay as mentioned by NCCLS.^[46]^

While determining the activity, Ampicillin and Griseofulvin were used as reference compounds for antibacterial and antifungal activity, respectively. The results are tabulated in Tables 2.

**Table 2:** Minimum inhibitory concentration [μg/mL] of Synthesized Compounds **3 a**–**j** and standard drugs against pathogenic microbes.

Gram −ve bacteria			Gram +ve bacteria		Fungi		
Compound	*E. coli* *MTCC 443*	*P. aeruginosa* *MTCC1688*	*S. aureus* *MTCC96*	*S.pyogenes* *MTCC442*	*C. albicans* *MTCC227*	*A. niger* *MTCC 282*	*A. clavatus* *MTCC1323*
**3 a**	100	**50**	**62.5**	125	500	>1000	>1000
**3 b**	125	250	**125**	100	500	500	500
**3 c**	100	250	**62.5**	250	**250**	500	1000
**3 d**	250	250	**125**	250	500	1000	>1000
**3 e**	**62.5**	100	250	250	**250**	1000	1000
**3 f**	250	500	**100**	250	500	250	500
**3 g**	100	250	**125**	500	1000	>1000	>1000
**3 h**	100	**62.5**	**100**	125	500	1000	>1000
**3 i**	**62.5**	125	**50**	**62.5**	500	1000	1000
**3 j**	100	125	**50**	250	**250**	500	500
**Ampicillin**	**100**	**100**	**250**	**100**			
**Griseofulvin**					**500**	**100**	**100**

The antibacterial screening results revealed that against *S. aureus*, all the tested derivatives displayed excellent inhibitory potential with MIC ranging 50 to 250 μg/mL when compared to standard drug Ampicillin (MIC=250 μg/mL). Compounds **3 i** and **3 j** were found most active with MICs of 50 g/mL while compounds **3 a** and **3 c** also demonstrated more potency than standard drug with MICs of 62.5 g/mL. Remaining compounds of series were also found to be more active than standard except **3 e** which showed equipotency. Against *E. coli*, compounds **3 e** and **3 i** have showed better inhibition than standard with MIC of 62.5 μg/mL, whereas compounds **3 a**, **3 c**, **3 g**, **3 h** and **3 j** demonstrated equipotent inhibition with MIC of 100 μg/mL. Tested compounds were found to present selective inhibition against *P. aeruginosa*. Compounds **3 a** and **3 h** were found more potent with MIC values of 50 and 62.5 μg/mL, respectively, while compound **3 e** found equipotent in comparison to standard with MIC of 100 μg/mL. The remaining compounds were found to show mild activity against *P. aeruginosa* pathogens. Only compounds **3 b** and **3 i** found active among the tested derivatives against *S. pyogenus*.

The comparative analysis of antibacterial potential of compounds **3 a**–**3 j** against all the tested strains is depicted in Graph 1. 
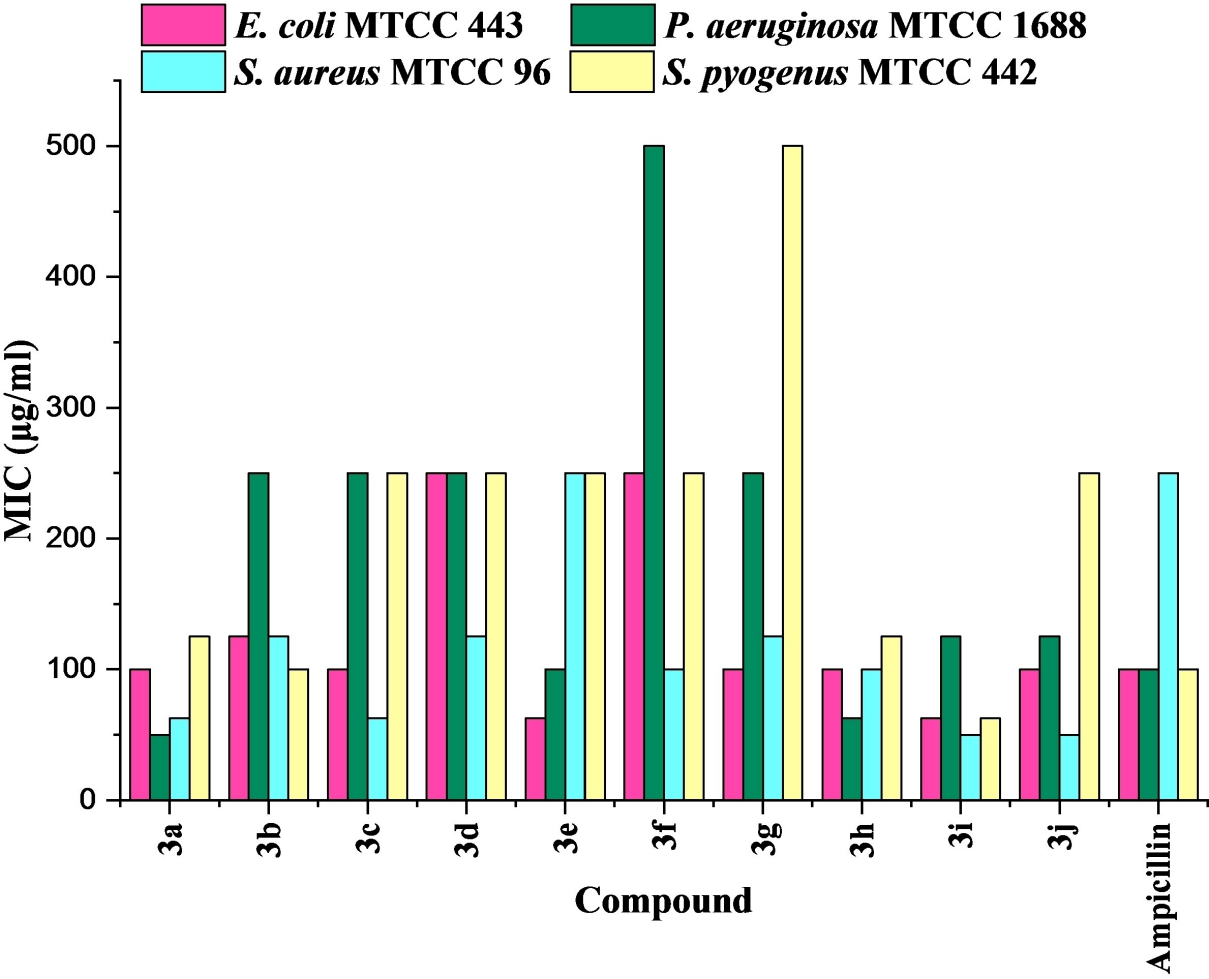


**Graph 1**. Comparative analysis of antibacterial potential of 2‐(1‐pyrazolyl)thiazoles.

Antifungal outcomes demonstrated that all the screened compounds were found to show promising results against *C. albicans* only. Compounds **3 c**, **3 e** and **3 j** displayed two fold increase in potency with MIC value of 250 μg/mL whereas other derivatives were found equipotent with MIC 500 μg/mL, except **3 g**, as compared to standard Griseofulvin. All the screened compounds exhibited moderate to mild activity against *A. niger* and *A. clavatus* fungal strains. Compounds **3 g** was found inactive against all three fungal strains. Only compound **3 f** showed moderate activity against *A. niger* with MIC of 500 μg/mL. Compound **3 b** & **3 j** showed mild activity against *A. niger* and *A. clavatus*.

#### 2.3.2 Anthelmintic Activity

The synthesized 5‐acylfunctionalized 2‐(*1H*‐pyrazol‐1‐yl)thiazoles **3 a**–**j** were evaluated for their *in vitro* anthelmintic activity against adult Indian earthworms, using Albendazole as reference drug. Earthworm species; *Pheretima. posthuma* was selected due to its anatomical and physiological similarity with the intestinal roundworm parasite of human and was procured from department of agriculture, Gurukul, Kurukshetra. DMSO was used as diluents to get desired concentration of compounds and activity was performed according to standard method. The experiment was performed at concentration 0.2 (% *w/v*) and in triplet for mean paralysis and death time. The death time was confirmed by placing earthworms in water at 50 °C which cause movement if worm was still alive. The results are summarized in Table 3.

**Table 3:** Anthelmintic activity of synthesized compounds **3 a**–**j**.

Sample code	Mean paralysis time (min)±SD	Mean death time (min)±SD
**3 a**	40.6±5.68	118±7.81
**3 b**	44.8±4.14	115.4±5.81
**3 c**	34.6±7.30	87.2±5.80
**3 d**	45.6±6.06	93.6±4.50
**3 e**	**25.4±1.14**	**78.6±6.99**
**3 f**	42.6±8.87	98.2±5.80
**3 g**	46.6±4.15	108±10.39
**3 h**	45.0±7.58	98.2±6.99
**3 i**	43.8±6.14	92±4.50
**3 j**	**30.8±3.96**	**77.8±7.91**
**Albendazole**	**29.0±0.8**	**71.2±4.92**

The results for anthelmintic screening revealed that all the synthesized compounds exerted mild to admirable anthelmintic activity compared with standard drug Albendazole. Compound **3 e** and **3 j** exhibited shortest mean paralysis time (25.4±1.14 min, 30.8±3.96 min) and mean death time (78.6±6.99 min, 77.8±7.91 min) respectively. While compound **3 g** exerted longest mean paralysis time (46.6±4.15 min) and mean death time (108±10.39 min) in comparison to standard used. The rest of compounds were found moderately active against tested earthworms.

### 2.4 *In Silico* Studies

#### 2.4.1 Molecular Docking Studies

Docking experiments were carried out to validate the examined biological activities of the synthesized analogues and further grasp the information about the mechanism by which they influenced their efficacy. The antibacterial target penicillin binding proteins 4 (PBP4) ***E. coli*** **(PDB ID: 2EX6)**,^[47]^ ***S. aureus*** **(PDB ID: 3HUN)** ^[48]^ that catalyze the final step of murein biosynthesis in bacteria and bacterial CD‐NTases ***P. aeruginosa*** **(PDB ID: 6P8U)**,^[49]^ Cas9 endonuclease protein ***S. pyogenes*** **(PDB ID: 4CMQ)** ^[50]^ that offer defence against invasive genetic elements like viruses and plasmids were selected as target receptor for molecular docking study because inhibition of these targets may help in the development of new antibacterial drugs.

The analysis of the docking results revealed that compounds displayed proficient binding at the active site of the receptors. All the compounds demonstrated binding energies in the range of −7.60 to −8.20 kcal mol^−1^ against gram positive *S. aureus*, −6.8 to −7.6 kcal mol^−1^ against gram positive *S. pyogenes* bacterial species. Likewise, all compounds exhibited binding energies in the range of −7.10 to −7.70 kcal mol^−1^ against gram negative *E. coli*, −6.0 to −7.6 kcal mol^−1^against gram negative *P. aeruginosa* bacterial species respectively. From the *in vitro* results, the most effective compounds **3 i** and **3 j** against *S. aureus*, **3 e** against *S. pyogenes*, **3 e** and **3 i** against *E. coli*, **3 a** and **3 h** against *P. aeruginosa* displayed comparable binding energies with the reference drug Ampicillin (Table 4). The binding energy analysis authenticated the better efficacy of synthesized compounds against the Gram positive bacterial species *S. aureus* compared with the rest of used gram positive *S. pyogenes* and gram negative *E. coli*, *P. aeruginosa* bacterial species.

**Table 4:** Binding Affinities (kcal mol^−1^) of target compounds **3 a**–**j** for *E. coli*, *P. aeruginosa*, *S. aureus*, *S. pyogenes* and *C. albicans*.

C. No.	*E. coli* (PDB ID: 2EX6)	*P. Aeruginosa* (PDB ID: 6P8U)	*S. aureus* (PDB ID: 3HUN)	*S. Pyogenes* (PDB ID: 4CMQ)	*C. albicans* (PDB ID: 5TZ1)
**3 a**	−7.20	−7.5	−7.60	−6.9	−6.90
**3 b**	−7.40	−7	−7.70	−7.4	−6.50
**3 c**	−7.40	−7.4	−8.00	−7.6	−7.10
**3 d**	−7.50	−6.9	−7.90	−7.6	−6.90
**3 e**	−7.40	−7.1	−8.00	−7.5	−7.80
**3 f**	−7.40	−7.1	−7.80	−7.1	−7.60
**3 g**	−7.20	−7.6	−7.70	−6.8	−7.00
**3 h**	−7.30	−7.3	−7.80	−7.3	−7.40
**3 i**	−7.70	−6.8	−8.00	−7.4	−7.00
**3 j**	−7.10	−6	−8.20	−7.2	−7.00
**Ampicillin**	−7.80	−7.7	−8.30	−7.9	
**Griseofulvin**					−7.0

**Figure 5:**
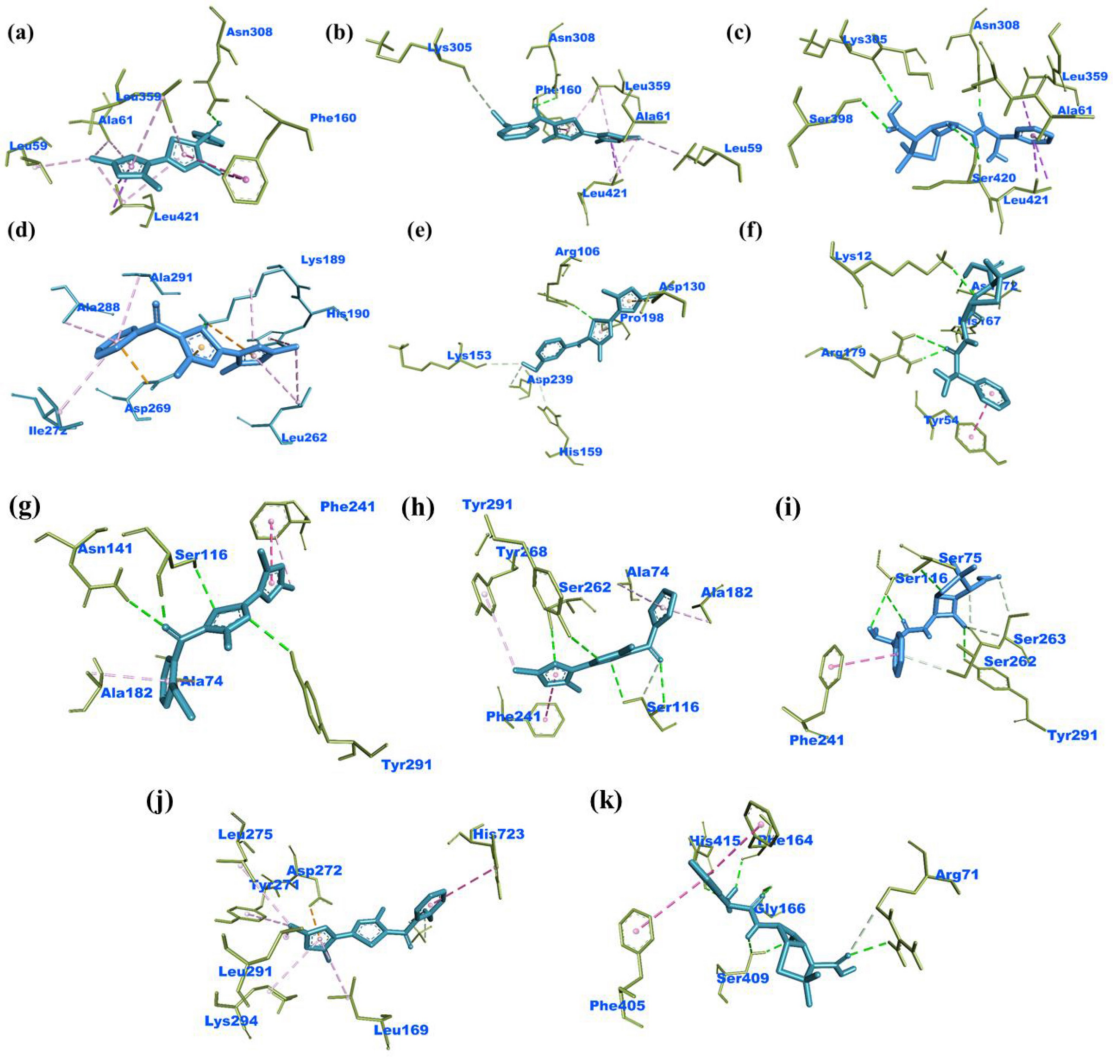
Binding interactions of **3 e** (a), **3 i** (b) and Ampicillin (c) with *E. coli* **(PDB ID: 2EX6); 3 a** (d), **3 h** (e), Ampicillin (f) with *P. aeruginosa* **(PDB ID: 6P8U); 3 i** (g), **3 j** (h), Ampicillin (i) with *S. aureus* **(PDB ID: 3HUN) and 3 i** (j), Ampicillin (k) with *S. pyogenes* **(PDB ID: 4CMQ)**.

The interactions with the most effective derivatives and standard drug Ampicillin against the bacterial strains were identified and it was observed that derivative **3 e** displayed interactions with the amino acid residues ALA^61^, LEU^359^, LEU^421^, ASN^308^ while **3 i** exhibited with LYS^305^, ASN^308^, ALA^61^, LEU^359^, LEU^421^ amino acid residues of the *E.coli* (**PDB ID: 2EX6**). The active compound **3 a** showed similar interactions with amino acid residues ALA^291^, ASP^269^, Leu^262^, HIS^190^ while compound **3 e** with ARG^106^, LYS^153^, HYS^159^, PRO^198^, ASP^239^ amino acids of *P. Aeruginosa* (**PDB ID: 6P8U**).The effective derivatives **3 j** interacted with the amino residues PHE^241^, SER^116^, SER^262^ while **3 i** with PHE^241^, TYR^291^, SER^116^ amino acids of *S. aureus* (**PDB ID: 3HUN**). The potent compound **3 i** was found to interact similarly with LEU^275^, TYR^271^, ASP^272^, HIS^723^, LEU^294^ amino acid residue of *S. Pyogenes* (**PDB ID: 4CMQ**). The Figure 5 depicted the interaction diagrams of the most effective antibacterial agents against used bacterial strains.

**Figure 6:**
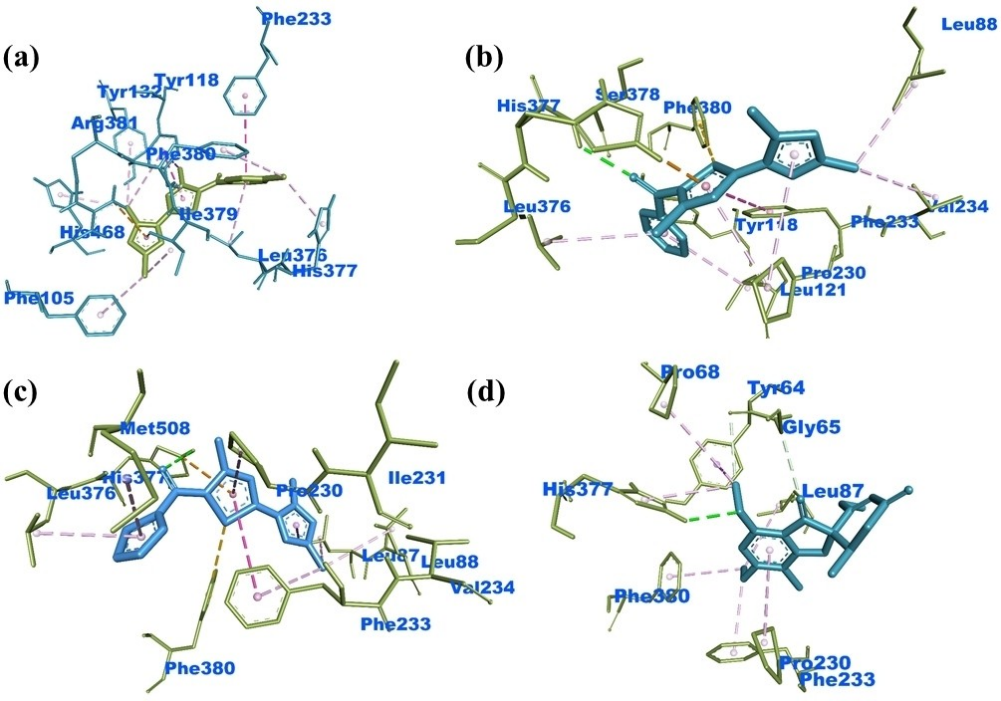
Binding interactions of compounds **3 c** (a), **3 e** (b), **3 j** (c) and Griseofulvin (d) with *C. albicans* **(PDB ID: 5TZ1)**.

The antifungal potency of the synthesized compounds was also investigated by their docking in the crystal structure of sterol 14‐alpha demethylase (CYP51) from *C. albicans* (**PDB ID: 5TZ1**). All the synthesized compounds depicted comparable binding energies with the reference antifungal drug **(**Table 4 **)**. The reference antifungal drug Griseofulvin interacted with the amino acid residues PRO^68^, TYR^64^, GLY^65^, LEU^87^, HIS^377^, PHE^386^, PRO^230^ and PHE^233^. Compound **3 c** showed conventional H‐bond with TYR^132^, π‐cation with ARG^381^, π‐donor H‐bond with TYR^118^, π‐σ with ILE^379^ amino acid residues. Compound **3 e** presented conventional H‐bonding with SER^378^, π‐cation with HIS^377^, π‐sulfur with PHE^380^ and other hydrophobic interactions. The compound **3 j** depicted conventional H‐bond with HIS^377^, π‐cation with HIS^377^, π‐sulfur with PHE^380^, π‐σ with LEU^87^ amino acid residues. These interactions were responsible for the prominent binding of the compounds in the binding cavity of the selected receptor. Figure 6 depicted the interaction diagrams of the most effective antifungal agents **3 c**, **3 e** and **3 j** against selected fungal strain.

#### 2.4.2 ADMET Properties

The journey of a drug molecule in the human body is investigated by investigating its Absorption, Distribution, Metabolism, Excretion and Toxicity (ADMET) properties. The experimental determination of these properties is expensive and time consuming. Thus, in order to reduce the cost and time, computational tools are employed to predict the drug like properties of the synthesized compounds. The analysis of the computed pharmacokinetic parameters revealed that all the compounds obeyed Lipinski rules of five (RO5). The values of the parameters were in the optimum range *i. e*. molecular weight (*≤*500 g mol^−1^), Log S (*≤*6), topological polar surface area (20–130 Å^2^), number of H‐bond acceptors and donors less than 10 and 5, respectively.^[51, 52]^ The toxicity studies of the derivatives indicated that all the derivatives (except **3 f**) belong to toxicity class IV i. e. harmful if swallowed. All the derivatives displayed inactivity in terms of nephrotoxicity, cardiotoxicity, immunotoxicity, cytotoxicity, and nutritional toxicity. The predicted results are shown in Table 5.

**Table 5:** ADMET properties of synthesized compounds **3 a**–**j**.

Molecule	**3 a**	**3 b**	**3 c**	**3 d**	**3 e**	**3 f**	**3 g**	**3 h**	**3 i**	**3 j**
MW	297.37	315.37	331.82	366.26	376.27	311.40	327.40	327.40	327.40	303.40
#Rotatable bonds	3	3	3	3	3	3	4	4	4	3
#H‐bond acceptors	3	4	3	3	3	3	4	4	4	3
#H‐bond donors	0	0	0	0	0	0	0	0	0	0
MR	84.01	83.97	89.02	94.03	91.71	88.97	90.50	90.50	90.50	81.89
TPSA	76.02	76.02	76.02	76.02	76.02	76.02	85.25	85.25	85.25	104.26
iLOGP	3.25	3.35	3.21	3.62	3.32	3.51	3.50	3.54	3.14	2.97
GI absorption	High	High	High	High	High	High	High	High	High	High
log Kp (cm/s)	−5.18	−5.22	−4.94	−4.71	−5.17	−5.00	−5.39	−5.39	−5.39	−5.20
Lipinski #violations	0	0	0	0	0	0	0	0	0	0
Ghose #violations	0	0	0	0	0	0	0	0	0	0
Veber #violations	0	0	0	0	0	0	0	0	0	0
Egan #violations	0	0	0	0	0	0	0	0	0	0
Muegge #violations	0	0	0	1	0	0	0	0	0	0
LD_50_ (mg/kg)	600	600	600	600	600	300	600	600	600	600
Toxicity Class	4	4	4	4	4	3	4	4	4	4
Hepatotoxicity	+	+	+	+	+	+	+	+	+	‐
Nephrotoxicity	–	–	–	–	–	–	–	–	–	–
Cardiotoxicity	–	–	–	–	–	–	–	–	–	–
Carcinogenicity	+	–	–	–	–	+	+	+	+	+
Immunotoxicity	–	–	–	–	–	–	–	–	–	–
Mutagenicity	+	+	+	+	+	+	–	–	–	+
Cytotoxicity	–	–	–	–	–	–	–	–	–	–
Clinical toxicity	+	+	–	–	–	+	+	+	+	–
Nutritional toxicity	–	–	–	–	–	–	–	–	–	–

*+for active and – for inactive.

**Figure 7:**
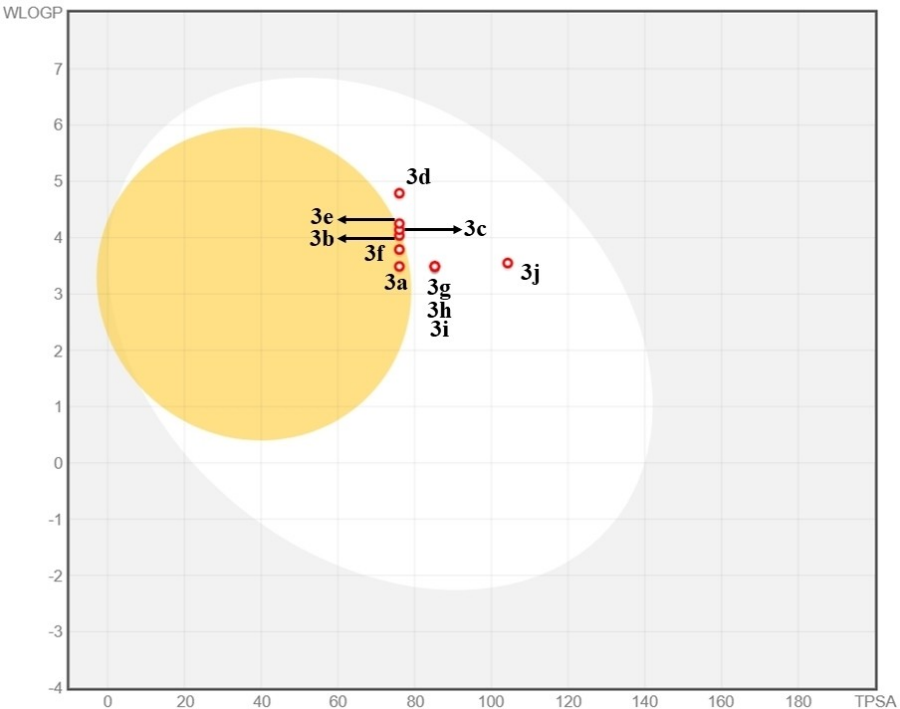
The blood brain barrier permeability (BBBP) and gastrointestinal absorption (GIA) properties of the compounds **3 a**–**j** using Brain or Intestinal estimated permeation predictive method.

In order to have good biological activity, a drug must have sufficient lipophilic nature to cross cell membrane and proper aqueous solubility for oral availability. These pharmacokinetic parameter blood brain barrier permeability (BBBP) and gastrointestinal absorption (GIA) properties of the examined derivatives were predicted with the boiled egg (Brain or Intestinal estimated) permeation method.^[53]^ The compounds **3 d**, **3 h**, **3 g**, **3 i** and **3 j** found in the white region of the egg and confirmed the high GI absorption of these compounds, while the compounds **3 a**, **3 b**, **3 c**, **3 e**, and **3 f** found in the yellow region of the egg showed their passive BBB permeation nature. Furthermore, all the compounds investigated in this examination were non‐substrates of Permeability‐glycoprotein (PGP−) which is important to estimate efflux through membrane as revealed by the red dots in the egg diagram Figure 7. The results clearly indicated that target compounds have excellent ADMET profile and could be potential drug candidate.

## 3 SAR analysis of 5‐acylfunctionalized 2‐(*1H*‐pyrazol‐1‐yl)thiazoles

1. Bioactive studies revealed that all the synthesized derivatives **3 a–j** displayed potent bioactivity against gram‐positive bacterial strains *S. aureus* with MIC values ranging from 50–250 μg/mL.
2. Introduction of *p*‐OMePh (**3 g**) favoured the antibacterial activity. Change in the position of OMe from *para* to *meta* and *ortho* position of phenyl ring strengthened the antibacterial potency e. g. 2‐OMe Ph (**3 i**) substitution showed five‐fold stronger potency against *S. aureus* than the standard drug and also showed admirable antimicrobial potential against *E. coli*, *P. aeruginosa* and *S. pyogenes* making it effective and broad spectrum antibacterial candidate. These results were substantiated by 2D plots of docking studies between **3 g**, **3 h**, **3 i** and *S. aureus* **(PDB ID: 3HUN)** [supplementary file, Figure S_1_–S_3_] which revealed that derivative **3 i** binds the receptor site with higher affinity via four conventional H‐bonding interactions in comparison to **3 g** and **3 h** which bind through three and two H‐bonding interactions respectively.
3. The incorporation of halogen Cl **(3 c)** and Br (**3 e**) at *para* position of phenyl ring and compound bearing thienyl ring (**3 j**) displayed excellent antifungal activity against *C. albicans*. Docking study revealed that these groups interacted efficiently with amino acid residues of selected target *via* conventional H‐bonding and hydrophobic interactions which is contributing factor for good activity.
4. Compound **3 d** and **3 f** bearing the substituents 2,4‐diClPh, 4‐MePh diminished the antimicrobial potential. Their low binding energy compared to standard drug revealed that these groups might be involved in poor interaction with amino acid residues present in the binding site of target protein.
5. Compound with *p*‐Br substitution on phenyl ring (**3 e**) and compound bearing thienyl ring (**3 j**) enhanced the anthelmintic activity against earthworms *Pheretima. Posthuma*.

**Figure 8:**
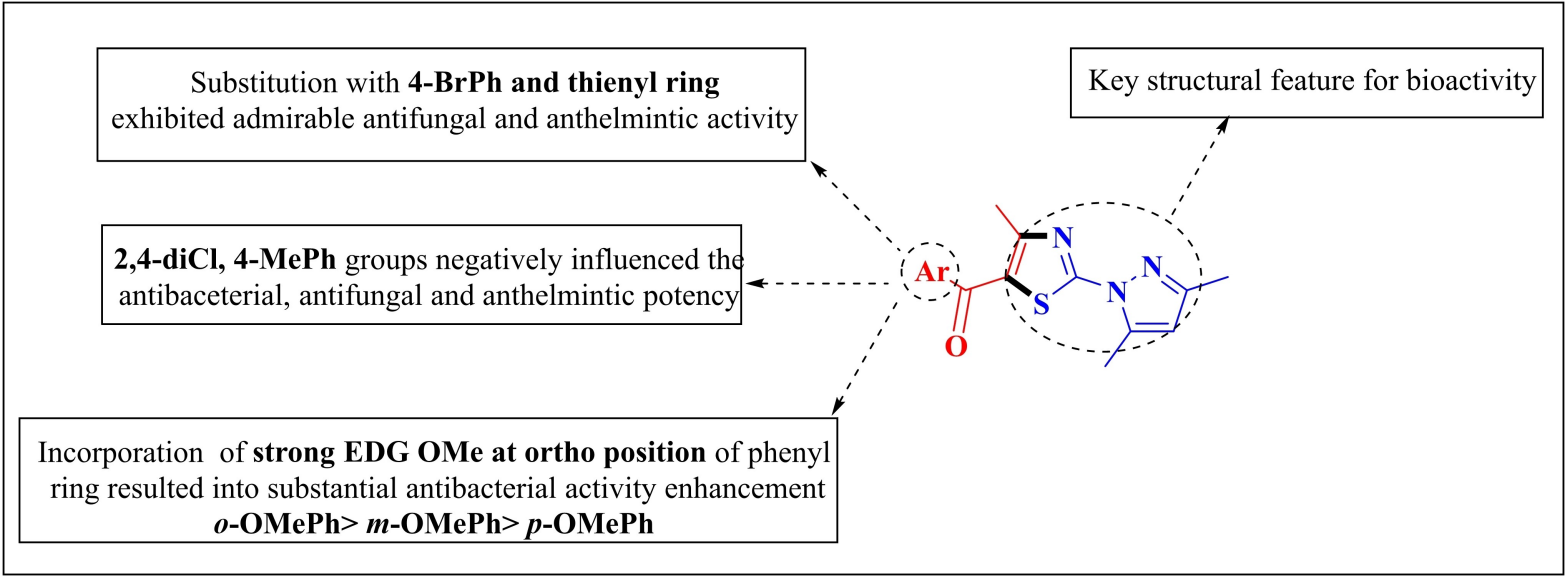
Structure‐activity relationship of 5‐acylfunctionalized 2‐(1*H*‐pyrazol‐1‐yl)thiazoles.

The SAR studies demonstrated that the variation in the type or position of the substituents has a major impact on the activity of the tested derivatives. Figure 8 represents a brief description of SAR.

## 4 Conclusions

In this study, we have designed and synthesized a series of 5‐acylfunctionalized 2‐(1*H*‐pyrazol‐1‐yl)thiazoles by integrating the pyrazole core and the thiazole ring according to the principle of combining pharmacophores and evaluated them for their antibacterial potential against two Gram‐positive (*S. aureus*, *S. pyogenusa*), two Gram‐negative bacterial strains (*E. coli*, *P. aeruginosa*) and three fungal species (*C. albicans*, *A. Niger*, *A. clavatus*). All the tested compounds **3 a**–**j** found to show excellent antimicrobial activity against *S. aureus*. Compounds **3 e** and **3 i** displayed more potency against *E. coli* in addition; **3 i** also displayed inhibition of *S. pyogenus*. Whereas compounds **3 a** and **3 h** showed more potency against *P. aeruginosa* than the used standard drug. All synthesized compounds were found active against *C. albicans* with equi/more potency than standard, except **3 g**. Also, compounds **3 e** and **3 j** were found to exhibit promising anthelmintic results. Furthermore, molecular docking studies were also in consistence with *in vitro* results and revealed significant interactions of active compounds within the active site of receptors. These results clearly indicated that the newly synthesized 5‐acylfunctionalized 2‐(*1H*‐pyrazol‐1‐yl)thiazole derivatives exhibit excellent antimicrobial activities and could serve as potent lead in development of new antimicrobial drug.

## Experimental Section

### General

All the chemicals and solvents used for the present study were purchased from commercial suppliers of Hi‐media and Avera, India, and used without purification. To monitor the progress of the reaction and purity of the products, thin layer chromatography (Merck silica gel 60 F254‐coated aluminum plates) was used. The mixture of ethyl acetate and pet ether solvents was used as the mobile phase and visualized under UV light at 254 nm. Melting points were determined using an electrical digital Melting Point Apparatus (MEPA) in an open capillary tube and were uncorrected. IR spectra were recorded on Buck Scientific IR M‐500 instrument in KBr pellets (υ_max_ in cm^−1^). The Jeol ECX‐500 spectrometer was used to record NMR spectra, using CDCl_3_ as a solvent and TMS as an internal standard (the chemical shift in δ ppm and coupling constants (*J*) were expressed in parts per million (ppm) and hertz, respectively). High‐resolution mass spectra (HRMS) were measured in the ESI+ mode at SAIF, Punjab University, Chandigarh and CIL, GJU, Hisar. 2D correlation spectroscopy, (^1^H−^13^C) gs‐HSQC, (^1^H−^13^C) gs‐HMBC, (^1^H−^15^N) gs‐HSQC and (^1^H−^15^N) gs‐HMBC of the samples were carried out at Kurukshetra University, Kurukshetra.

### Experimental Procedure for the Synthesis of 5‐acylfunctionalized 2‐(*1H*‐pyrazol‐1‐yl)thiazoles 3 a–j

β‐diketones **2** (0.162 g, 1.0 mmol) were ground with NBS (0.178 g, 1.0 mmol) for 5 min. Subsequently, 3,5‐dimethyl‐*1H*‐pyrazole‐1‐carbothioamide **1** (0.155 g, 1.0 mmol) was added and pulverized well in presence of sodium carbonate (0.0265 g, 0.025 mmol) for 40–45 minutes at 80–90 °C. After completion of the reaction, as monitored by TLC (ethyl acetate:petroleum ether; 80 : 20, *v/v*), the reaction mixture was cooled to room temperature, diluted with water and extracted with ethylacetate (2×25 mL). The combined organic extract was dried over Na_2_SO_4_ and concentrated. Further, the crude residue was recrystallized from EtOH and dried to yield pure pyrazolyl‐thiazoles **3** in high yields.

#### (2‐(3,5‐dimethyl‐1*H*‐pyrazol‐1‐yl)‐4‐methylthiazol‐5‐yl)(phenyl)methanone (3 a)

White solid; m. p. 146 °C; yield: 85 %; IR (KBr) ν_max_ (cm^−1^): 1628 (C=O); ^1^H NMR (400 MHz, CDCl_3_) δ=7.83 (m, 2H, 2′′,6′′‐H), 7.57 (m, 1H, 4′′‐H), 7.47 (m, 2H, 3′′,5′′‐H), 6.02 (s, 1H, 4′‐H), 2.69 (s, 3H, 5′‐CH_3_), 2.62 (s, 3H, 4‐CH_3_), 2.26 (s, 3H, 3′‐CH_3_); ^13^C NMR (100 MHz, CDCl_3_) δ=188.5, 163.7, 158.8, 152.6, 142.5, 139.9, 132.3, 128.6, 128.5, 125.4, 110.4, 18.8, 13.9, 13.6; HRMS (ESI): m/z calcd for C_16_H_15_N_3_OS: 297.0936; found: 298.0914 [M+1]^+^; Elemental analysis: Calcd. For C_16_H_15_N_3_OS: C, 64.62; H, 5.08; N, 14.13 % Found: C, 64.60; H, 5.03; N, 14.09 %.

#### (2‐(3,5‐dimethyl‐1*H*‐pyrazol‐1‐yl)‐4‐methylthiazol‐5‐yl)(4‐fluorophenyl)methanone (3 b)

White solid; m. p. 111–113 °C; yield: 86 %; IR (KBr) ν_max_ (cm^−1^): 1643 (C=O); ^1^H NMR (400 MHz, CDCl_3_) δ=7.88 (m 2H, 2′′,6′′‐H), 7.15 ( m, 2H, 3′′,5′′‐H), 6.02 (s, 1H, 4′‐H), 2.69 (s, 3H, 5′‐CH_3_), 2.61 (s, 3H, 4‐CH_3_), 2.26 (s, 3H, 3′‐CH_3_); ^13^C NMR (100 MHz, CDCl_3_) δ=187.0, 166.6, 164.1, 163.8, 159.0, 152.8, 142.6, 136.22, 136.19, 131.46, 131.37, 125.1, 115.8, 115.6, 110.6, 18.9, 14, 13.7; HRMS (ESI): m/z calcd for C_16_H_14_N_3_FOS: 315.0842; found 316.0801 [M+1]^+^; Elemental analysis: Calcd. For C_16_H_14_N_3_FOS: C, 60.94; H, 4.47; N, 13.32 % Found: C, 60.92; H, 4.44; N, 13.27 %.

#### (4‐chlorophenyl)(2‐(3,5‐dimethyl‐1H‐pyrazol‐1‐yl)‐4‐methylthiazol‐5‐yl)methanone (3 c)

White solid; m. p. 137–140 °C; yield: 88 %; IR (KBr) ν_max_ (cm^−1^): 1643 (C=O); ^1^H NMR (400 MHz, CDCl_3_) δ=7.79 (d, *J*=6.8 Hz, 2H, 2′′,6′′‐H), 7.45 (d, *J*=6.8 Hz, 2H, 3′′,5′′‐H), 6.02 (s, 1H, 4′‐H), 2.69 (s, 3H, 5′‐CH_3_), 2.61(s, 3H, 4‐CH_3_), 2.26 (s, 3H, 3′‐CH_3_); ^13^C NMR (100 MHz, CDCl_3_) δ=187.1, 163.8, 159.2, 152.8, 142.6, 138.7, 138.2, 130.1, 128.8, 124.9, 110.5, 18.9, 13.9, 13.6; HRMS (ESI): m/z calcd for C_16_H_14_N_3_ClOS: 331.0546; found: 332.0627 [M+1]^+^, 334.0571 [M+1+2]^+^; Elemental analysis: Calcd. For C_16_H_14_N_3_ClOS: C, 57.92; H, 4.25; N, 12.66 % Found: C, 57.89; H, 4.21; N, 12.63 %.

#### (2,4‐dichlorophenyl)(2‐(3,5‐dimethyl‐1H‐pyrazol‐1‐yl)‐4‐methylthiazol‐5‐yl)methanone (3 d)

White solid; m. p. 118–120 °C; yield: 91 %; IR (KBr) ν_max_ (cm^−1^): 1636 (C=O); ^1^H NMR (400 MHz, CDCl_3_) δ=7.46 (m, 1H, 6′′‐H), 7.35 (s, 2H, 3′′,5′′‐H), 6.00 (s, 1H, 4′‐H), 2.67 (s, 3H, 5′‐CH_3_), 2.53 (s, 3H, 4‐CH_3_), 2.25 (s, 3H, 3′‐CH_3_); ^13^C NMR (100 MHz, CDCl_3_) δ=185.7, 165.0, 159.8, 153.1, 142.6, 138.5, 136.7, 131.6, 130.1, 128.9, 127.4, 127.2, 110.8, 18.5, 13.9, 13.6; HRMS (ESI): m/z calcd for C_16_H_13_Cl_2_N_3_OS: 365.0156; found: 366.0123 [M+1]^+^, 368.0112 [M+1+2]^+^, 370.0134 [M+1+4]^+^; Elemental analysis: Calcd. For C_16_H_13_Cl_2_N_3_OS: C, 52.47; H, 3.58; N, 11.47 % Found: C, 52.42; H, 3.53; N, 11.42 %.

#### (4‐bromophenyl)(2‐(3,5‐dimethyl‐1H‐pyrazol‐1‐yl)‐4‐methylthiazol‐5‐yl)methanone (3 e)

White solid; m. p. 119–121 °C; yield: 90 %; IR (KBr) ν_max_ (cm^−1^): 1643 (C=O); ^1^H NMR (400 MHz, CDCl_3_) δ=7.71 (d, *J*=6.8 Hz, 2H, 2′′,6′′‐H), 7.62 (d, *J*=6.8 Hz, 2H, 3′′,5′′‐H), 6.02 (s, 1H, 4′‐H), 2.69 (s, 3H, 5′‐CH_3_), 2.61(s, 3H, 4‐CH_3_), 2.26 (s, 3H, 3′‐CH_3_); ^13^C NMR (100 MHz, CDCl_3_) δ=187.2, 163.8, 159.3, 152.8, 142.5, 138.6, 131.7, 130.2, 127.3, 124.9, 110.5, 18.9, 13.9, 13.6; HRMS (ESI): m/z calcd for C_16_H_14_BrN_3_OS: 375.0041; found: 376.0099 [M+1]^+^; 378.0077 [M+1+2]^+^; Elemental analysis: Calcd. For C_16_H_14_BrN_3_OS: C, 51.07; H 3.75; N, 11.17 % Found: C, 51.02; H, H 3.69; N, 11.12 %.

#### (2‐(3,5‐dimethyl‐1H‐pyrazol‐1‐yl)‐4‐methylthiazol‐5‐yl)(p–tolyl)methanone (3 f)

Brown solid; m. p. 116–118 °C; yield: 79 %; IR (KBr) ν_max_ (cm^−1^): 1632 (C=O); ^1^H NMR (400 MHz, CDCl_3_) δ=7.75 (d, 2H, *J*=6.4 Hz, 2′′,6′′‐H), 7.26 (d, 2H, *J*=6.4 Hz, 3′′,5′′‐H), 6.00 (s, 1H, 4′‐H), 2.68 (s, 3H, 5′‐CH_3_), 2.60 (s, 3H, 4‐CH_3_), 2.43 (s, 3H, 4′′‐ CH_3_), 2.26 (s, 3H, 3′‐CH_3_); ^13^C NMR (100 MHz, CDCl_3_) δ=188.2, 163.5, 158.3, 152.5, 143.2, 142.4, 137.1, 129.1, 128.9, 125.5, 110.3, 21.6, 18.8, 13.9, 13.6; HRMS (ESI): m/z calcd for C_17_H_17_N_3_OS: 311.1092; found: 312.1078 [M+1]^+^; Elemental analysis: Calcd. For C_17_H_17_N_3_OS: C, 65.57; H 5.50; N, 13.49 % Found: C, 65.51; H, H 5.18; N, 13.41 %.

#### (2‐(3,5‐dimethyl‐1H‐pyrazol‐1‐yl)‐4‐methylthiazol‐5‐yl)(4‐methoxyphenyl)methanone (3 g)

Brown solid; m. p. 119–121 °C; yield: 79 %; IR (KBr) ν_max_ (cm^−1^): 1634 (C=O); ^1^H NMR (400 MHz, CDCl_3_) δ=7.87 (d, 2H, *J*=9.2 Hz, 2′′,6′′‐H), 6.95 (d, 2H, *J*=9.2 Hz, 3′′,5′′‐H), 6.01 (s, 1H, 4′‐H), 3.89 (s, 3H, 4′′‐OCH_3_), 2.69 (s, 3H, 5′‐CH_3_), 2.59 (s, 3H, 4‐CH_3_), 2.27 (s, 3H, 3′‐CH_3_); ^13^C NMR (100 MHz, CDCl_3_) δ=187.2, 163.3, 157.8, 152.5, 142.5, 132.4, 131.4, 125.5, 113.8, 110.4, 55.5, 18.7, 13.9, 13.6; HRMS (ESI): m/z calcd for C_17_H_17_N_3_O_2_S: 327.1041; found: 328.1018 [M+1]^+^; Elemental analysis: Calcd. For C_17_H_17_N_3_O_2_S: C, 62.37; H 5.23; N, 12.83 % Found: C, 62.30; H, 5.18; N, 12.76 %.

#### (2‐(3,5‐dimethyl‐1H‐pyrazol‐1‐yl)‐4‐methylthiazol‐5‐yl)(3‐methoxyphenyl)methanone (3 h)

Brown solid; m. p. 76–78 °C; yield: 82 %; IR (KBr) ν_max_ (cm^−1^): 1634 (C=O); ^1^H NMR (400 MHz, CDCl_3_) δ=7.42 (dt, 1H, *J_1_* =7.6 Hz, *J_2_* =1.4 Hz, 5′′‐H), 7.37 (t, 1H, *J*=8 Hz, 6′′‐H), 7.33( dd, 1H, *J_1_* =2.6 Hz, *J_2_* =1.4 Hz, 2′′‐H), 7.10 (ddd, 1H, *J_1_* =8 Hz, *J_2_* =2.8 Hz, *J_3_* =1.2 Hz, 4′′‐H), 6.0 (s, 1H, 4′‐H), 3.86 (s, 3H, 3′′‐OCH_3_), 2.69 (s, 3H, 5′‐CH_3_), 2.62 (s, 3H, 4‐CH_3_), 2.26 (s, 3H, 3′‐CH_3_); ^13^C NMR (100 MHz, CDCl_3_) δ=188.3, 163.91, 159.7, 152.7, 142.6, 141.1, 129.6, 125.49 121.2, 118.8, 113.1, 110.5, 55.5, 19, 14.0, 13.7; HRMS (ESI): m/z calcd for C_17_H_17_N_3_O_2_S: 327.1041; found: 328.1018 [M+1]^+^; Elemental analysis: Calcd. For C_17_H_17_N_3_O_2_S: C, 62.37; H 5.23; N, 12.83 % Found: C, 62.32; H, 5.18; N, 12.78 %.

#### (2‐(3,5‐dimethyl‐1H‐pyrazol‐1‐yl)‐4‐methylthiazol‐5‐yl)(2‐methoxyphenyl)methanone (3 i)

Brown solid; m. p. 86–88 °C; yield: 81 %; IR (KBr) ν_max_ (cm^−1^): 1634 (C=O); ^1^H NMR (400 MHz, CDCl_3_) δ=7.42 (dt, 1H, *J_1_* =8 Hz, *J_2_* =2.8, 6′′‐H), 7.37 (t, 1H, *J*=8 Hz, 4′′‐H), 7.33 (dd, 1H*, J_1_* =2.4 Hz, *J_2_* =1.6 Hz, 5′′‐H), 7.10 (ddd, *J_1_* =8 Hz, *J_2_* =2.4 Hz, *J_3_* =1.2 Hz, 1H, 3′′‐H), 6.01 (s, 1H, 4′‐H), 3.86 (s, 3H, 2′′‐OCH_3_); 2.69 (s, 3H, 5′‐CH_3_), 2.62 (s, 3H, 4‐CH_3_), 2.26 (s, 3H, 3′‐CH_3_); ^13^C NMR (100 MHz, CDCl_3_) δ=188.3, 163.9, 159.7, 159.0, 152.7, 142.6, 141.3, 129.6, 125.49, 121.2, 118.8, 113.1, 110.5, 55.5, 19.0, 14.0, 13.7; HRMS (ESI): m/z calcd for C_17_H_17_N_3_O_2_S: 327.1041; found: 328.1018 [M+1]^+^;Elemental analysis: Calcd. For C_17_H_17_N_3_O_2_S: C, 62.37; H 5.23; N, 12.83 % Found: C, 62.33; H, H 5.16; N, 12.72 %.

#### (2‐(3,5‐dimethyl‐1H‐pyrazol‐1‐yl)‐4‐methylthiazol‐5‐yl)(thiophen‐2‐yl)methanone (3 j)

Black solid; m. p. 116–118 °C; yield: 87 %; IR (KBr) ν_max_ (cm^−1^): 1634 (C=O); ^1^H NMR (400 MHz, CDCl_3_) δ=7.92 (dd, 1H, *J_1_* =4 Hz, *J_2_* =1.2 Hz, 5′′‐H); 7.69 (dd, 1H, *J_1_* =4 Hz, *J_2_* =1.2 Hz, 3′′‐H), 7.15 (m, 1H, 4′′‐H), 6.03 (s, 1H, 4′‐H), 2.70 (s, 6H, 4, 5′‐ CH_3_), 2.29 (s, 3H, 3′‐CH_3_); ^13^C NMR (100 MHz, CDCl_3_) δ=178.8, 159.4, 152.6, 145.4, 142.6, 133.9,132.9, 128.0, 123.1, 110.5, 18.8, 13.6; HRMS (ESI): m/z calcd for C_14_H_13_N_3_OS_2_: 303.0500; found: [M+1]^+^; 304.0474 [M+1]^+^; Elemental analysis: Calcd. For C_14_H_13_N_3_OS_2_: C, 55.42; H 4.32; N, 13.85 % Found: C, 55.37; H, H 4.28; N, 13.79 %.

### Biological Assay

#### Antimicrobial Activity Assay

The Gram‐positive and Gram‐negative bacterial strains consisted of *Staphylococcus aureus, Streptococcus pyogenusa, Escherichia coli, Pseudomonas aeruginosa* and fungal species consisted *Candida albicans, Aspergillus niger* and *Aspergillus clavatus*. All bacterial and fungal strains were procured from Institute of Microbial Technology, Chandigarh. Mueller Hinton Broth and Sabouraud dextrose broth used for bacterial and fungal nutrition, respectively.

The antimicrobial potential of newly synthesized derivatives was evaluated by broth dilution method. Mueller‐Hinton broth was used as nutrient medium to grow and dilute the compound suspension for the test bacteria. 2 % DMSO in water was used as the diluent to get desired concentration of compounds to test upon standard bacterial strains. Sabouraud Dextrose broth was used for fungal nutrition. Inoculum size for test strain was adjusted to 10^8^ Cfu mL^−1^ by comparing the turbidity. Serial dilutions were prepared in primary and secondary screening. Each synthesized compound and the standard drugs were diluted obtaining 2000 μg/mL concentration as a stock solution. The drugs which were found to be active in primary screening (i. e. 1000, 500 and 250 μg/mL concentrations) were further screened in their second set of dilution at 200, 100, 50, 25, 12.5 and 6.25 μg/mL concentration against all microorganisms. 10 μL suspensions were further inoculated on appropriate media and growth was noted after 24 and 48 hrs. The control tube containing no antibiotic was immediately sub cultured by spreading a loopful evenly over a quater of plate of medium suitable for the growth of the test organism. The tubes were then put for incubation at 37 °C overnight. The maximum dilution preventing appearance of turbidity was considered as minimal inhibitory concentration (MIC, μg/mL). All the tubes showing no visible growth (same as control tube) were subcultured and incubated overnight at 37 °C. The amount of growth from the control tube before incubation was compared. In this study Ampicillin was used as the standard antibacterial drugs while and Griseofulvin were used as standard antifungal drugs.

#### Anthelmintic Evaluation Assay

*In vitro* anthelmintic evaluation of all the synthesized 5‐acylfunctionalized 2‐(1‐pyrazolyl)thiazoles **3 a**–**j** were performed using Indian earthworms (*Pheretima posthuma*). Albendazole was selected as standard drug. Indian earthworms were collected from the agriculture department, Gurukul, Kurukshetra of almost equal size (4–5 cm in length and 0.1–0.2 cm in width). All earthworms washed with standard saline (0.9 % w/v) in order to remove dirt and fecal material. The reference drug and test compounds were dissolved in a DMSO (minimum quantity) and the solutions were adjusted to the volume to 20 mL by addition of standard saline (0.9 % NaCl). The assay was performed at concentrations 0.2 % (w/v) accordance the standard method^[54]^ with minor modification. The test concentrations were poured into two inches petri dishes. Six earthworms as a group were released into each of 20 mL of reference drug and the prepared test suspension at room temperature. The standard saline (0.9 % NaCl) solution with earthworms was served as a negative control for assay. The time taken by individual earthworm to get paralyzed and dead were observed up to 5 h. The paralysis time for worm was recorded as mean when no movement in worm was observed, even on shaking vigorously. Afterwards, the death time was also documented as mean *via* confirming that worm was not movable either on shaking vigorously or while given peripheral stimuli (hot water, 50 °C).^[55]^

#### Molecular Docking Details

The three dimensional crystal structures of two different bacterial strains, namely Gram positive *S. aureus* (**PDB ID: 3HUN**), *S. pyrogenes* **(PDB ID: 4CMQ)** and Gram negative *E. coli* (**PDB ID: 2EX6**), *P. aeruginosa* **(PDB ID: 6P8U)** were retrieved from RCSB data bank (https://www.rcsb.org/) and utilized for carrying out docking studies. The crystal structure of reference antifungal drug Griseofulvin against the *C. albicans* is not available. However, to identify possible mode of action of synthesized compounds, we selected the crystal structure of sterol 14‐alpha demethylase (CYP51) from *C. albicans* (**PDB ID: 5TZ1**) for carrying out docking studies.^[56]^ ChemDraw was used to sketch the structure of the molecules. Autodock vina^[57]^ software was employed for docking studies and auto dock tools was used for the protein and ligand preparation. The grid parameters for S. aureus were center_x=−20.622, center_y=1.096, center_z=0.232, size_x=54, size_y=34, size_z=36 and for E. coli were center_x=88.52, center_y=−4.459, center_z=43.862, size_x=34, size_y=26, size_z=32, respectively. The grid parameters for the P. aeruginosa were; center_x=−32.151, center_y=7.003, center_z=13.379, size_x=126, size_y=126, size_z=126 and for S. pyrogenes were center_x=−24.236, center_y=−23.697, center_z=−3.877, size_x=126, size_y=126, size_z=126, respectively.For theantifungal strain C. albicans, the grid parameters were center_x=62.710, center_y=66.776, center_z=4.109, size_x=126, size_y=126, size_z=126. For all the docking studies, the grid spacing was set to 0.375 Å, number of modes were 12 and exhaustiveness was set to 32. Discovery studio visualizer was used to analyse the outcomes of the docking process.

#### ADMET Prediction Details

In the present investigation, SwissADME^[58]^ (http://www.swissadme.ch/) and ProTox‐3.0^[59]^ (https://tox.charite.de/protox3/) were employed to compute the ADMET properties.

## 5 Funding Information

Council of Scientific and Industrial Research (CSIR), New Delhi, India

## Conflict of Interests

The author(s) declare no conflict of interest.

6

## Supporting information

As a service to our authors and readers, this journal provides supporting information supplied by the authors. Such materials are peer reviewed and may be re‐organized for online delivery, but are not copy‐edited or typeset. Technical support issues arising from supporting information (other than missing files) should be addressed to the authors.

Supporting Information

## Data Availability

The data that support the findings of this study are available in supplementary material of this article. Respected Sir, the above‐mentioned statement is our correct version of data availability statement.
