# Supplementary material for: Eco‐friendly Regioselective Synthesis, Biological Evaluation of Some New 5‐acylfunctionalized 2‐(1H‐pyrazol‐1‐yl)thiazoles as Potential Antimicrobial and Anthelmintic Agents
Source: ChemistryOpen. 2024 Aug 8;13(11):e202400142. doi: 10.1002/open.202400142 (PMC11564866; doi:10.1002/open.202400142)
Supplement: Supplementary file 1 — Supporting Information [file OPEN-13-e202400142-s001.pdf]

ChemistryOpen

Supporting Information

Eco-friendly Regioselective Synthesis, Biological Evaluation of Some New 5-acylfunctionalized 2-(*1H*-pyrazol-1-yl)thiazoles as Potential Antimicrobial and Anthelmintic Agents

Ranjana Aggarwal,* Manisha Sharma, Mona Hooda, Prabodh C. Sharma, and Diksha Sharma

SUPPLEMENTARY DATA

for

Eco-friendly Regioselective Synthesis, Characterization and Biological Evaluation of Some Novel 5- acylfunctionalized 2-(1-pyrazolyl)thiazoles as Potential Antimicrobial and Anthelmintic Agents

Ranjana Aggarwal,[a,b]* Manisha Sharma,[a] Mona Hooda,[c] Prabodh C. Sharma,[d] Diksha Sharma[e]

[a]Department of Chemistry, Kurukshetra University, Kurukshetra-136119, Haryana, India

[b]Council of Scientific and Industrial Research-National Institute of Science Communication and Policy Research, New Delhi 110012, India

[c]Department of Chemistry, Gurugram University, Gurugram-122003, Haryana, India

[d]School of Pharmaceutical Science, Delhi Pharmaceutical Science and Reasearch University, New Delhi 110017, India

[e]Swami Devi Dyal Institute of Pharmacy, Golpura, Barwala, 134118

*Corresponding author:

Prof. Ranjana Aggarwal, CSIR-National Institute of Science Communication and Policy Research, New Delhi, India. Tel: +91-9896740740

E-mails: *ranjana67in@yahoo.com*, *ranjanaaggarwal67@gmail.com*

TABLE of CONTENT

Contents

1. ^{1}H and ^{13}C NMR spectrum of title compounds (3a-j) S3-S14

2. 2D NMR HMBC, HSQC spectra and data table (S_1) of compound (3a) S15-S17

3. HRMS for the compounds (3a, 3c, 3e) S18-S20

4. 2D plots of ligand 3g, 3h and 3i interactions with S. aureus (PDB ID: 3HUN) S21

5-Benzoyl-4-methyl-2-(3,5-dimethyl-1*H*-pyrazolyl)thiazole (3a)

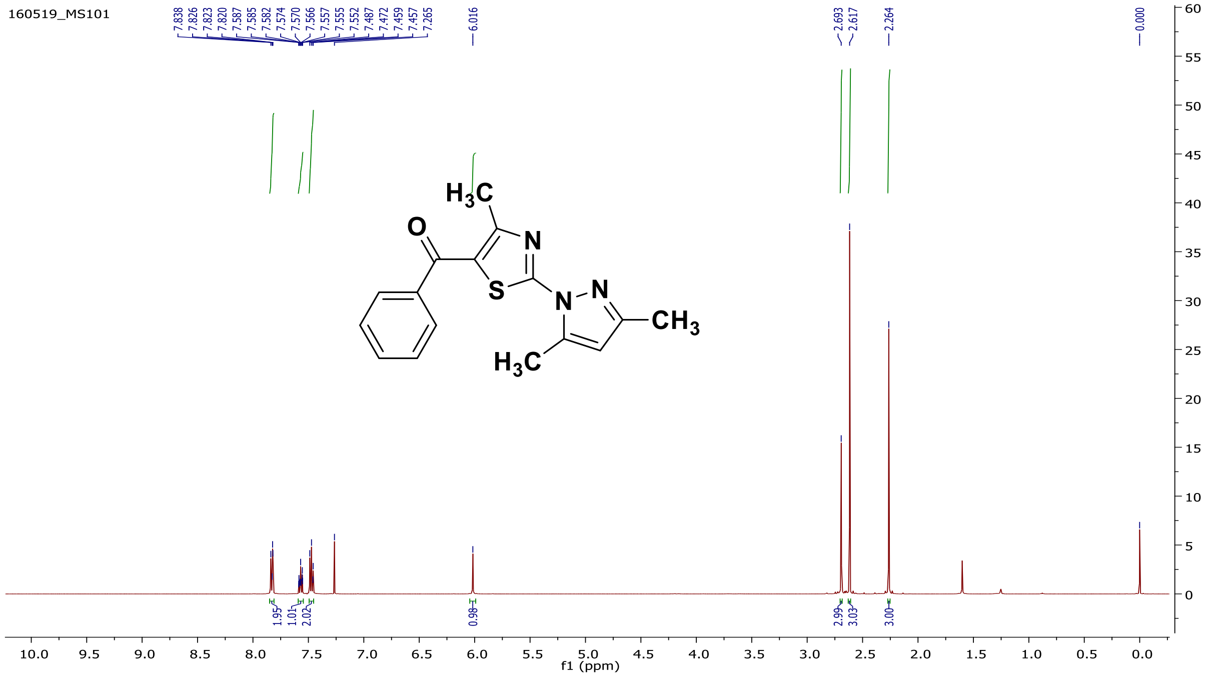

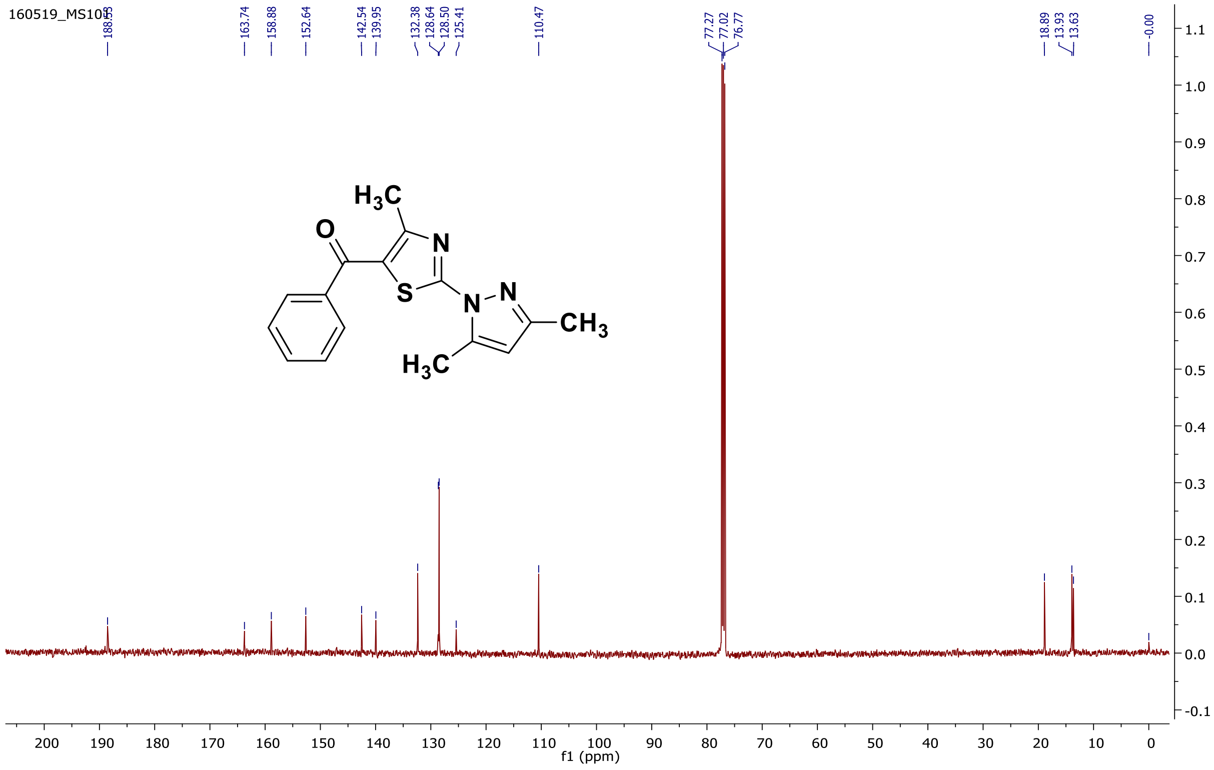

5-(4-Fluorobenzoyl)-4-methyl-2-(3,5-dimethyl-1*H*-pyrazolyl)thiazole (3b)

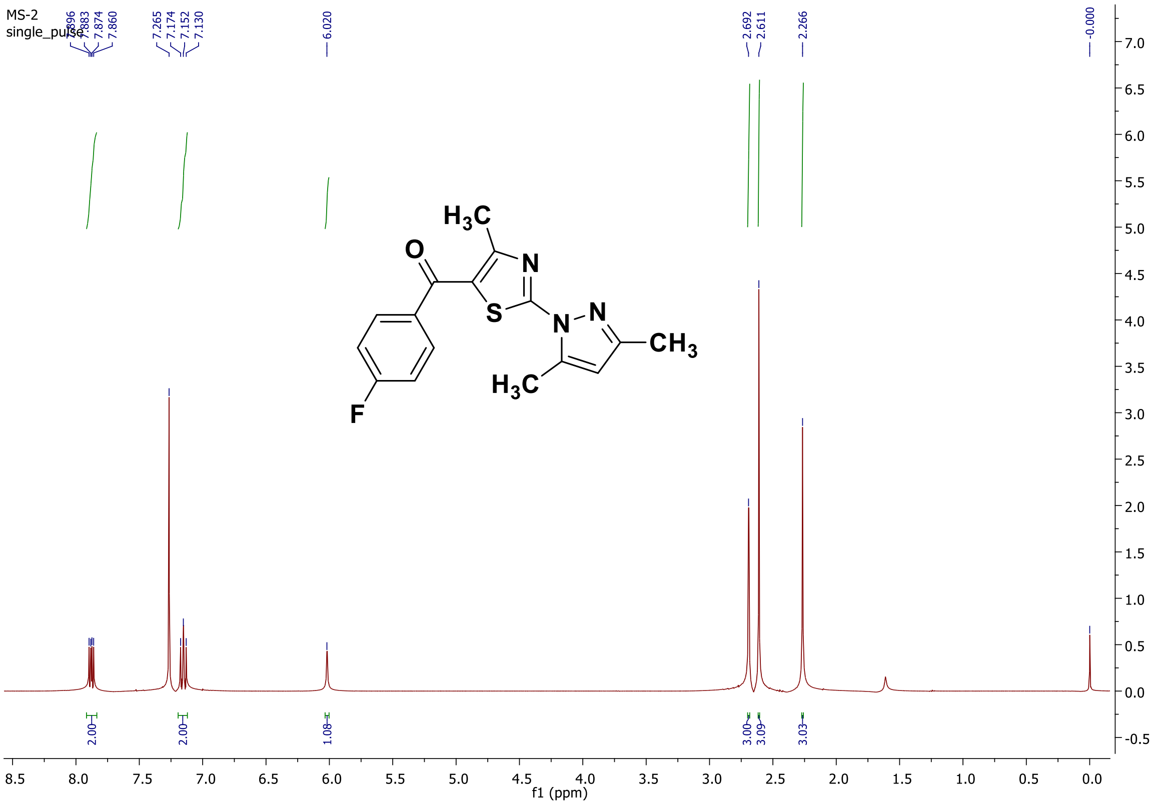

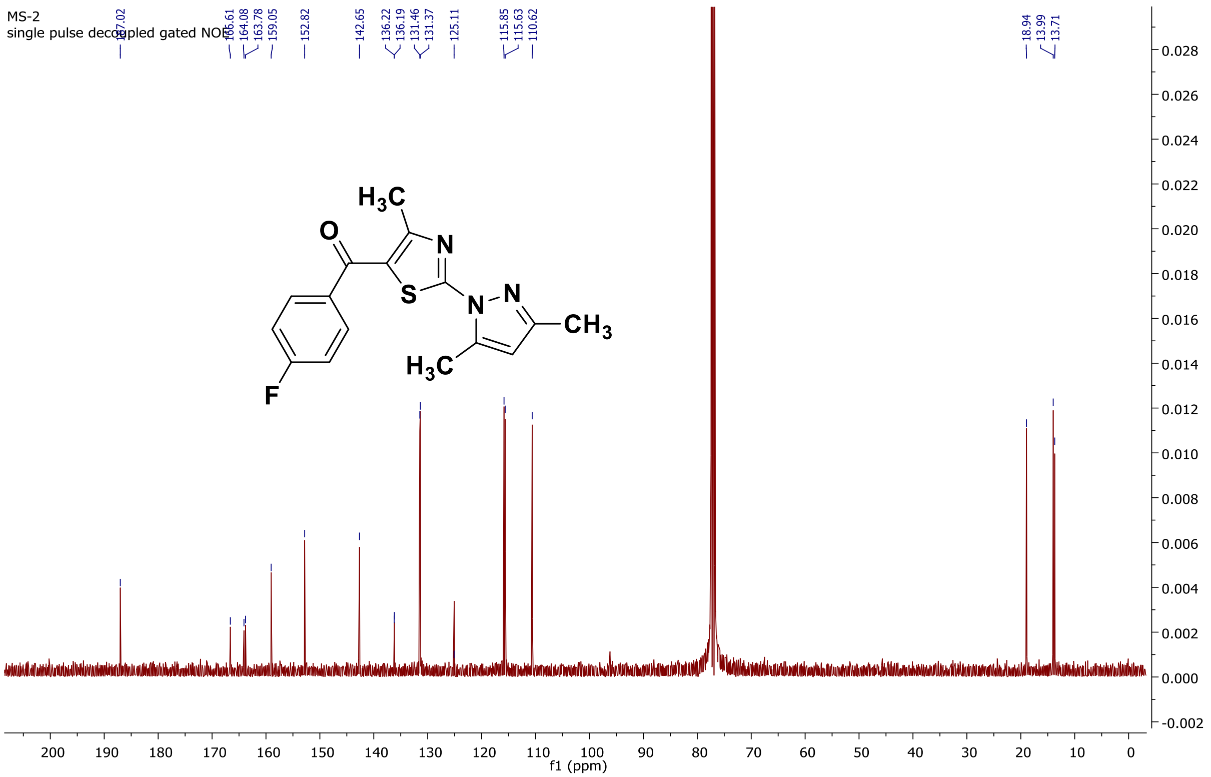

5-(4-Chlorobenzoyl)-4-methyl-2-(3,5-dimethyl-1*H*-pyrazolyl)thiazole (3c)

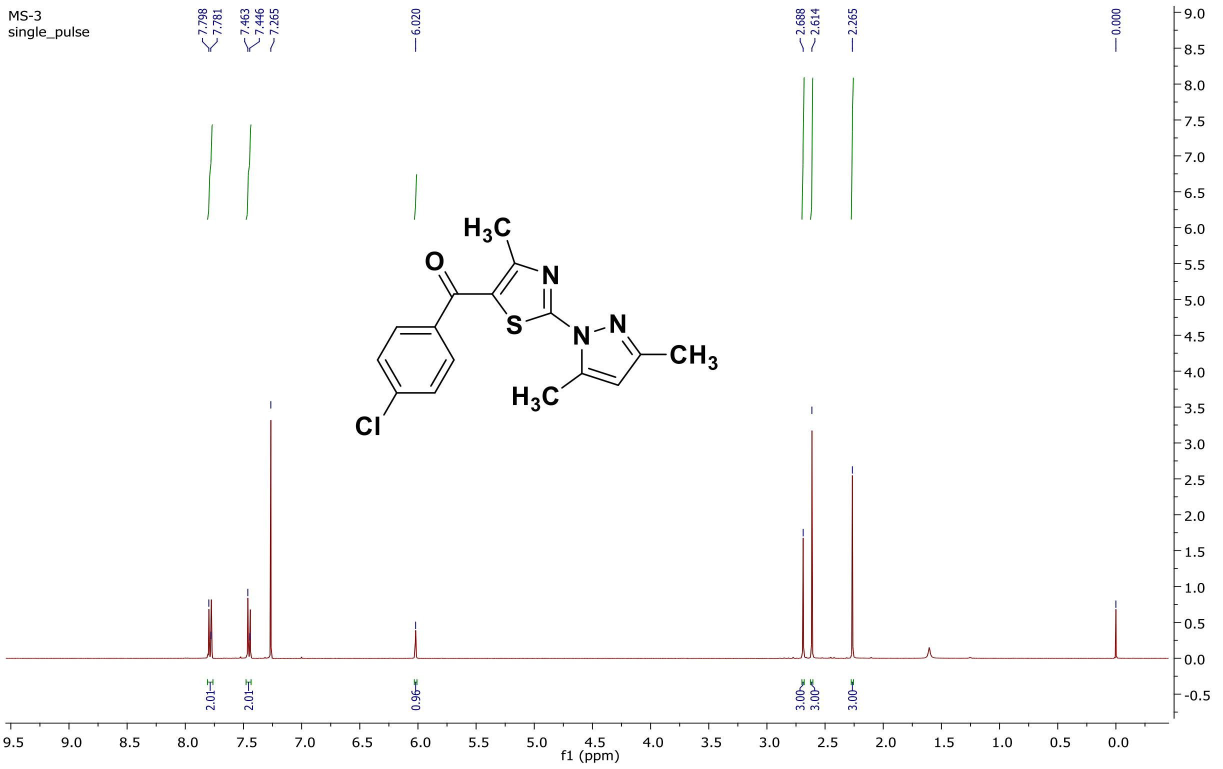

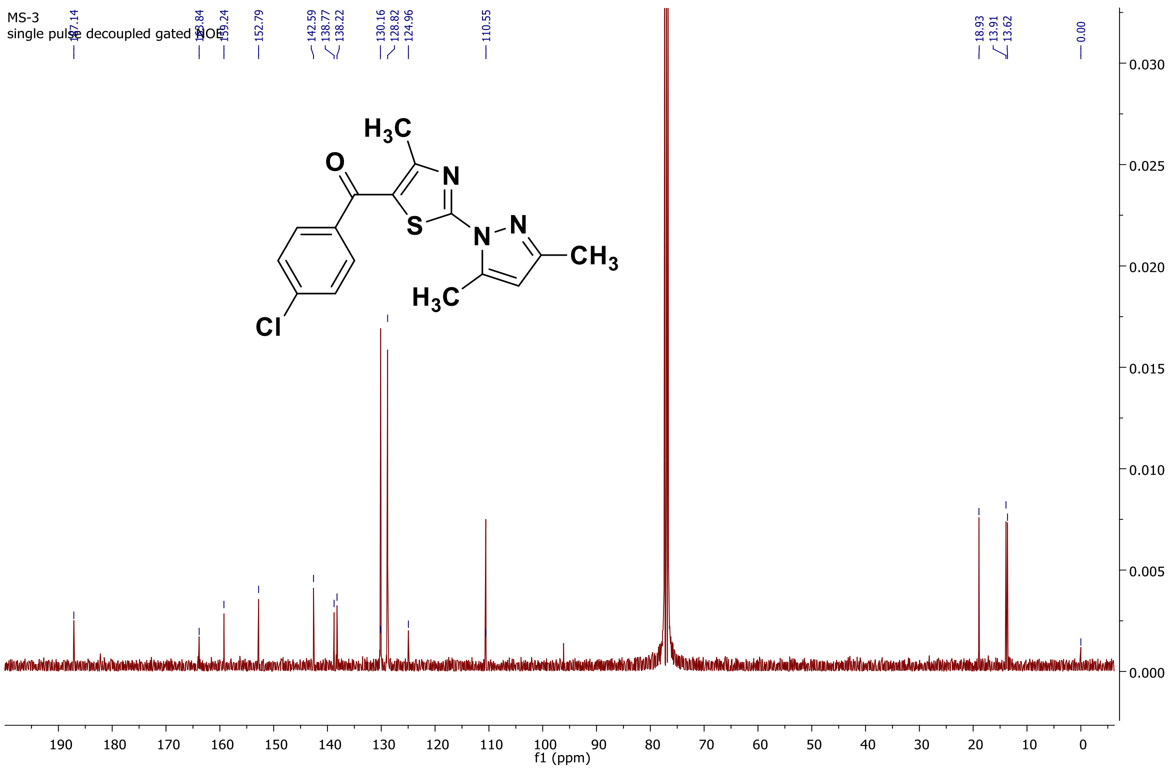

5-(2,4-Dichlorobenzoyl)-4-methyl-2-(3,5-dimethyl-1*H*-pyrazolyl)thiazole (3d)

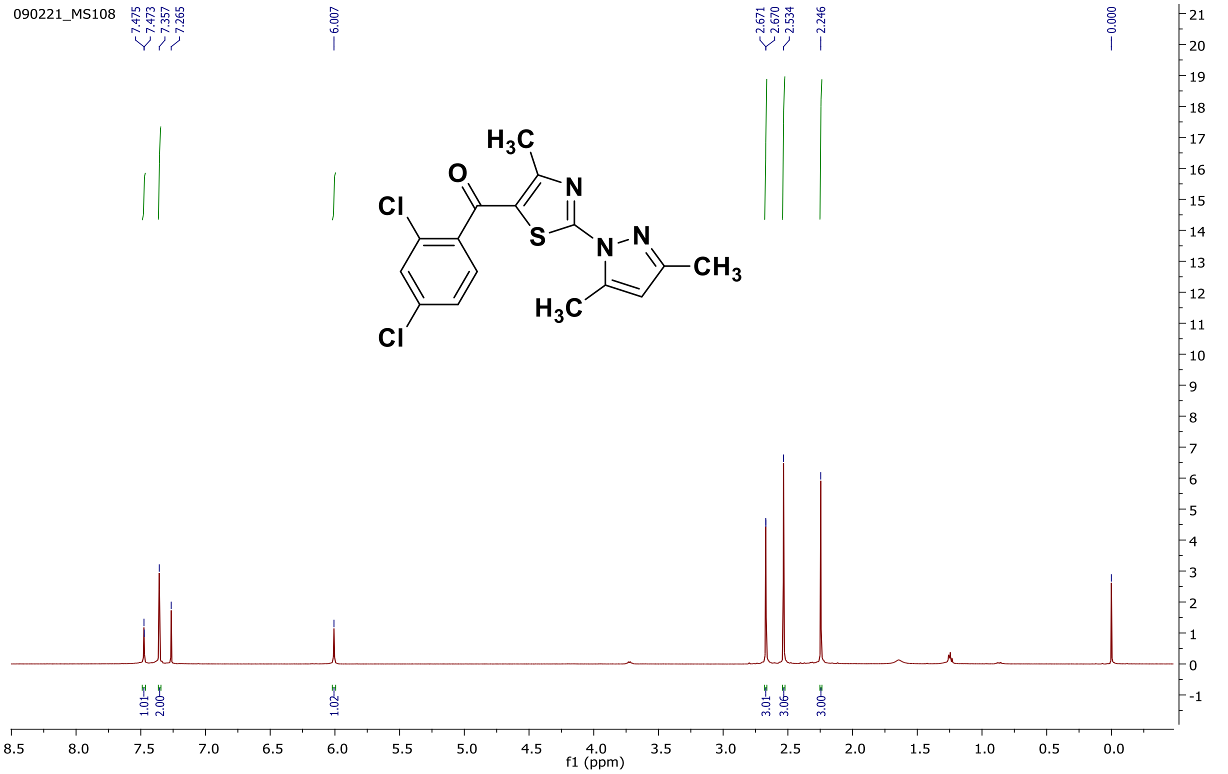

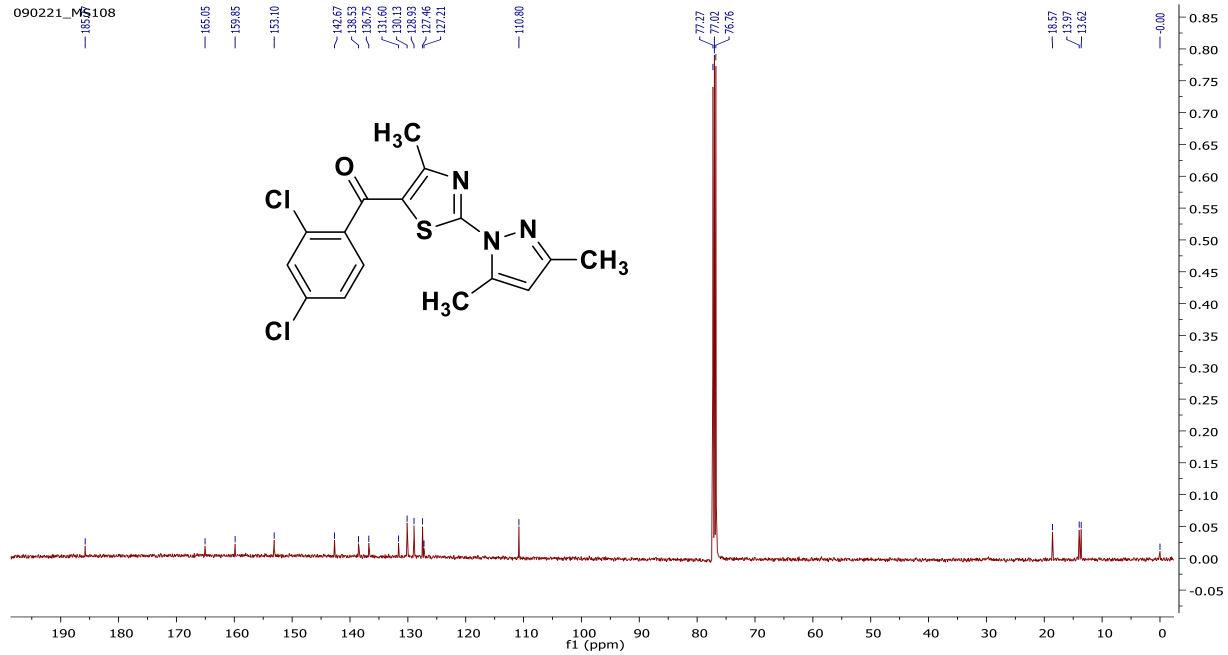

5-(4-Bromobenzoyl)-4-methyl-2-(3,5-dimethyl-1H-pyrazolyl)thiazole (3e)

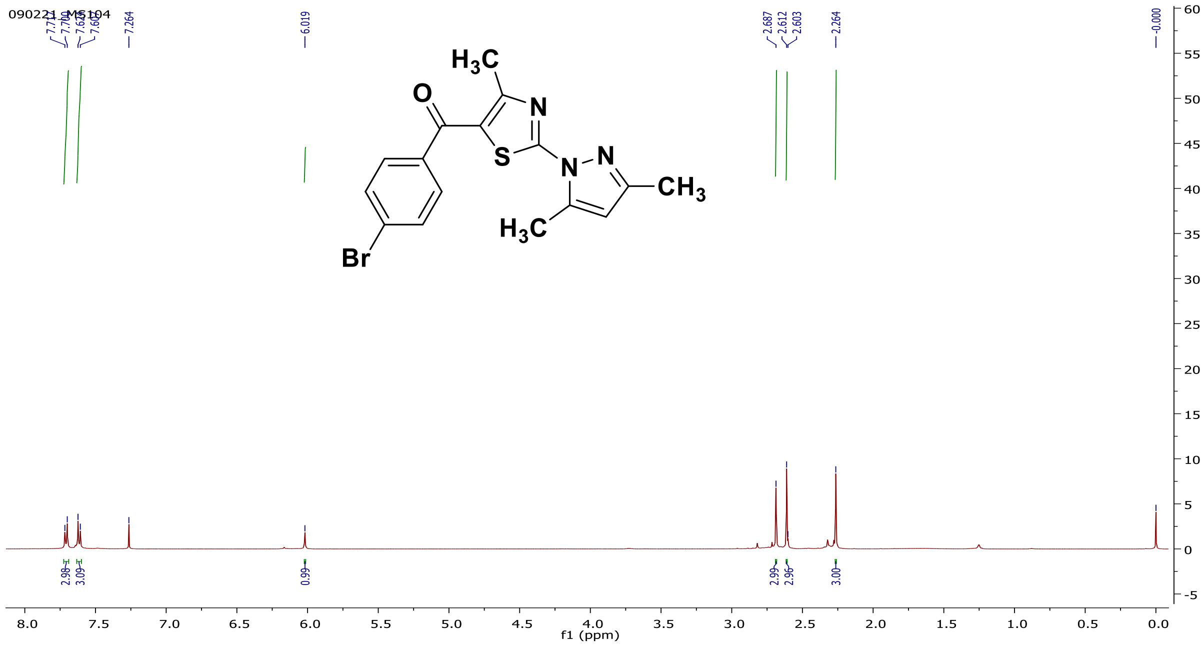

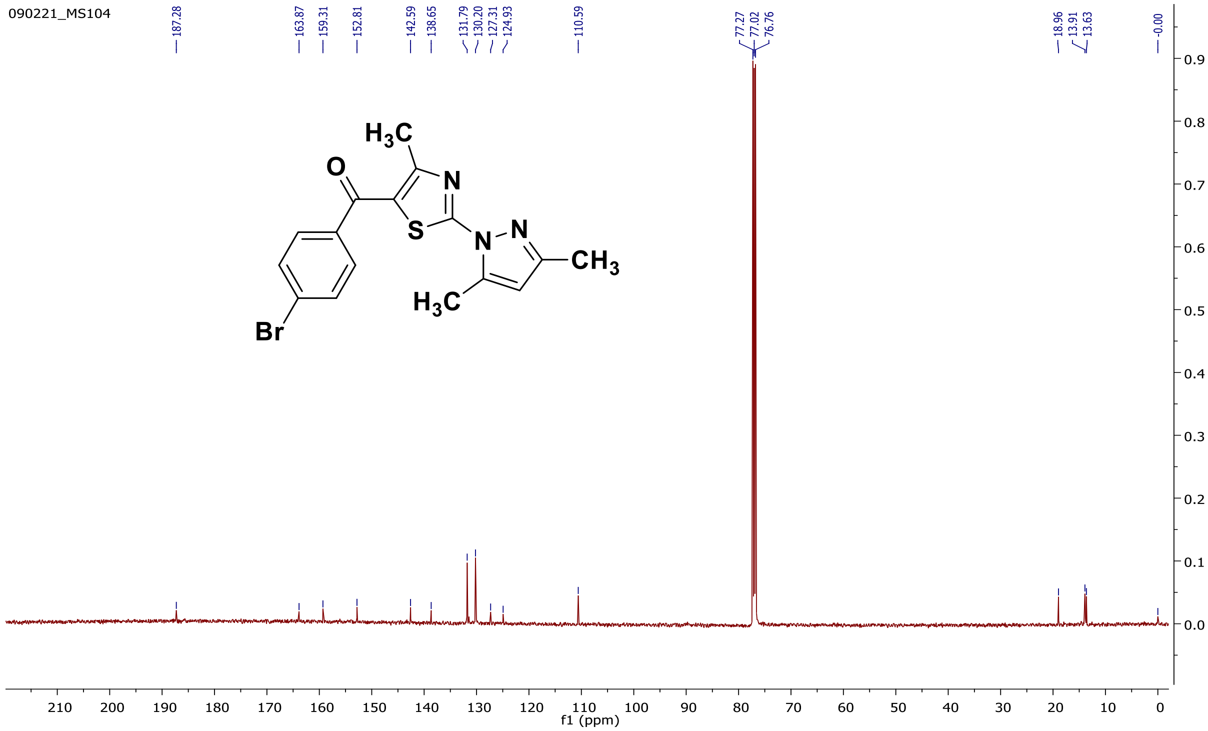

5-(4-Methylbenzoyl)-4-methyl-2-(3,5-dimethyl-1*H*-pyrazolyl)-thiazole (3f)

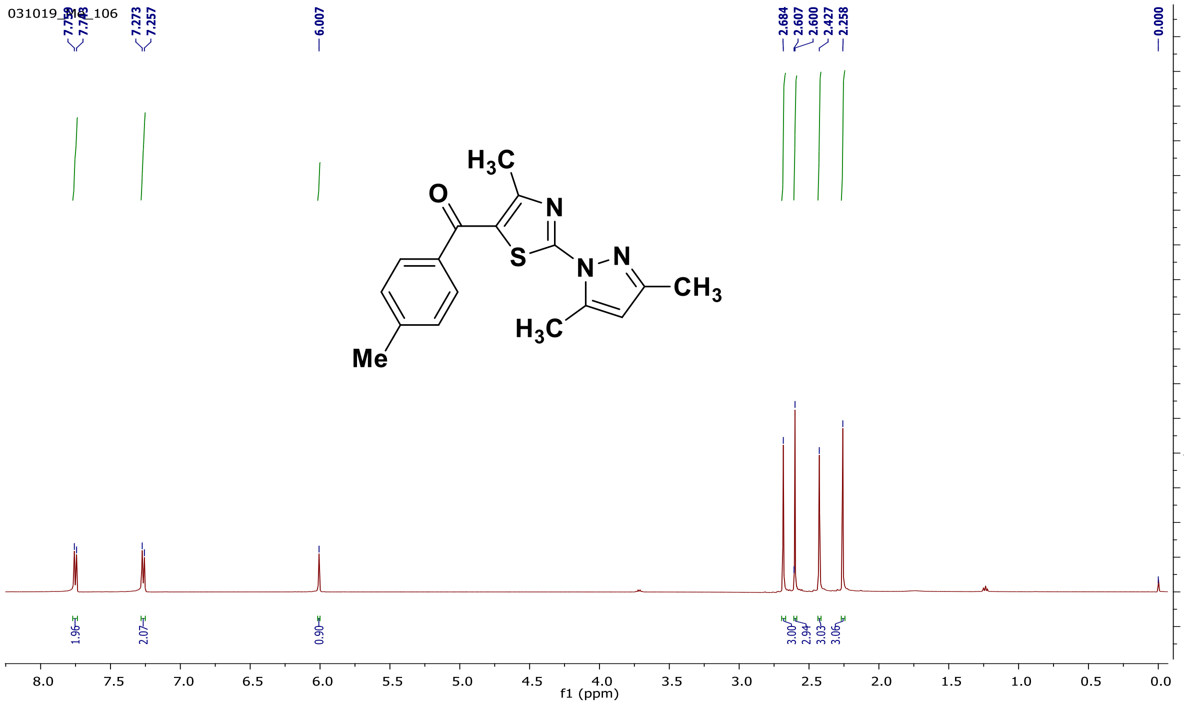

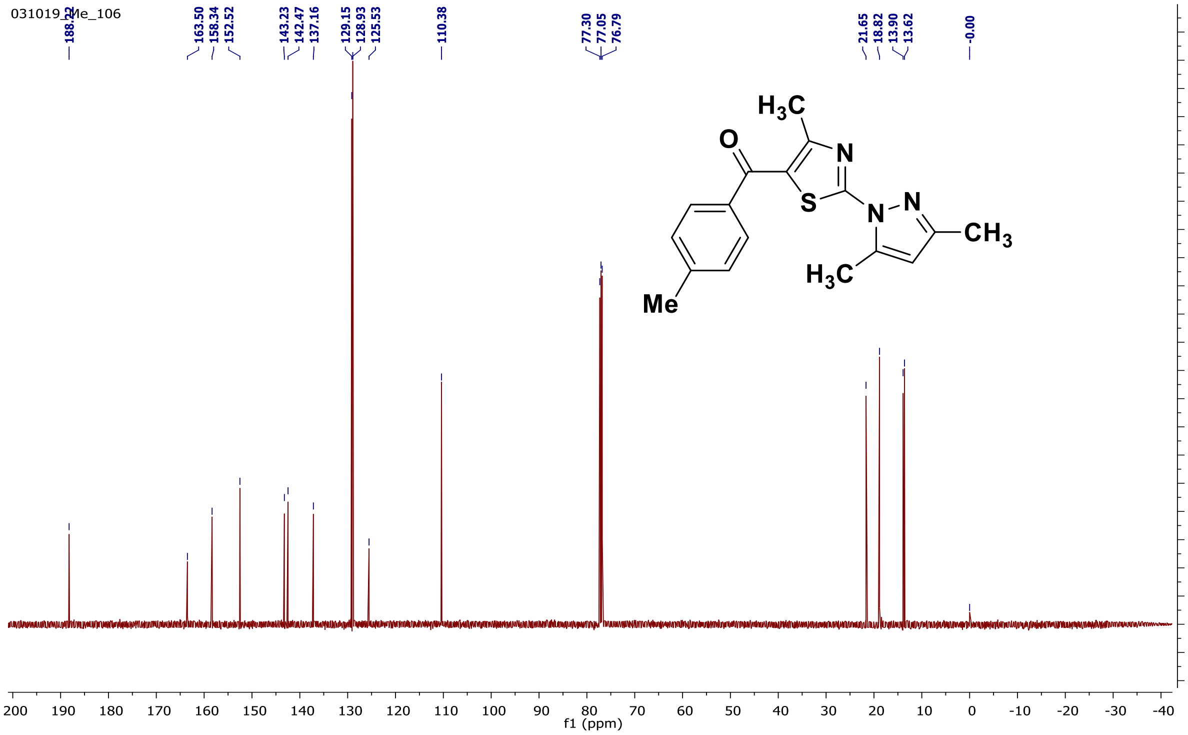

5-(4-Methoxybenzoyl)-4-methyl-2-(3,5-dimethyl-1*H*-pyrazolyl)thiazole (3g)

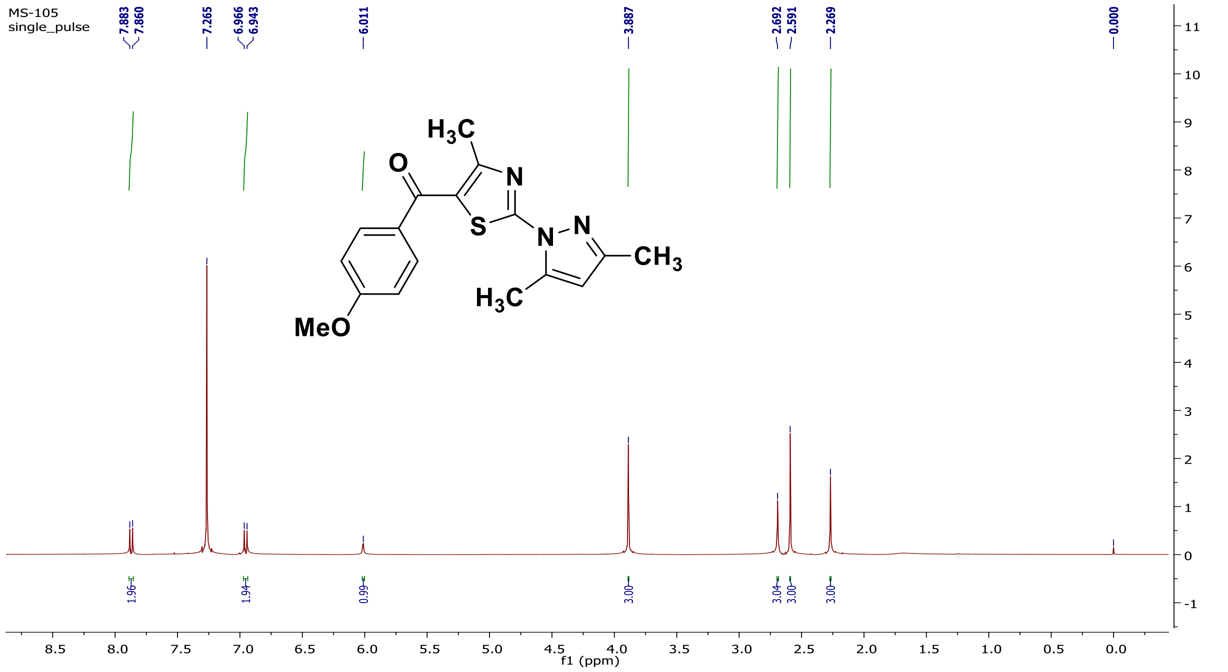

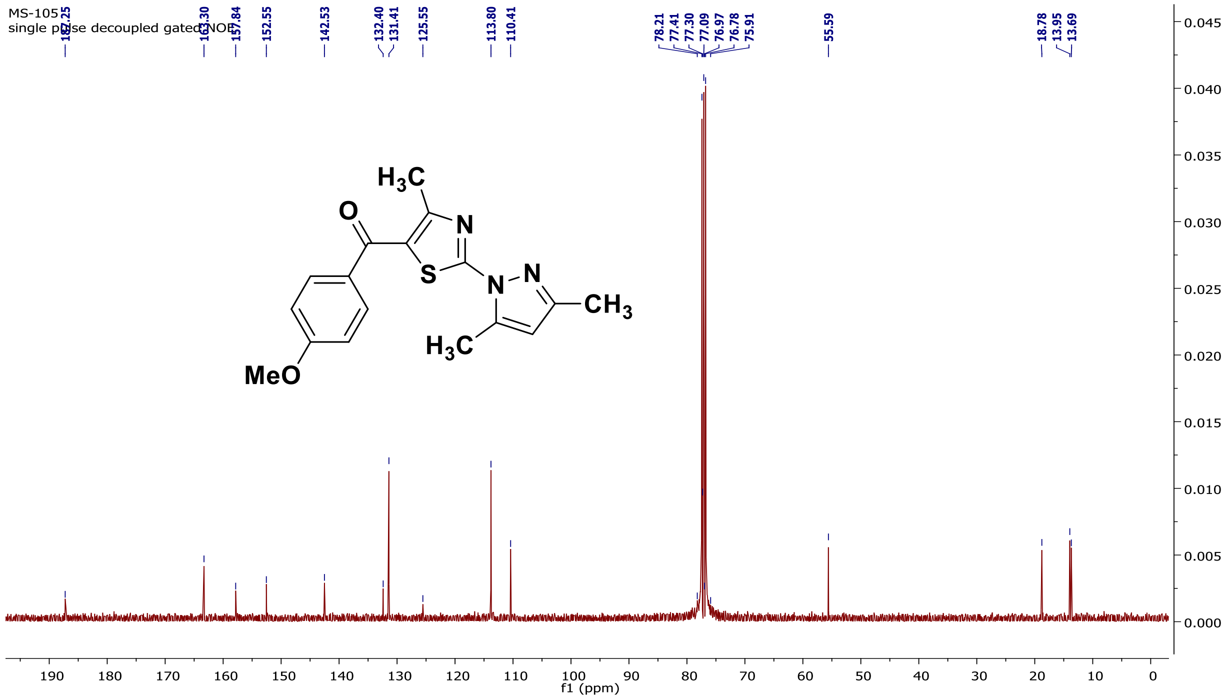

5-(3-Methoxybenzoyl)-4-methyl-2-(3,5-dimethyl-1*H*-pyrazolyl)thiazole (3h)

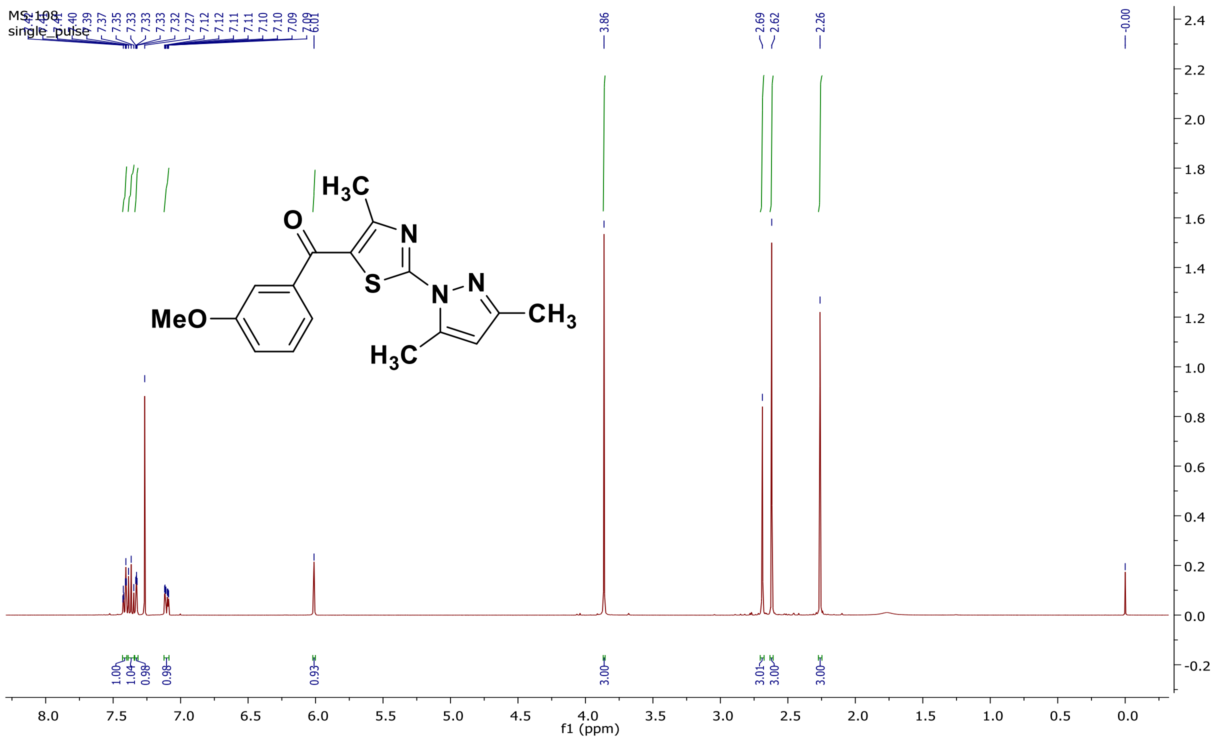

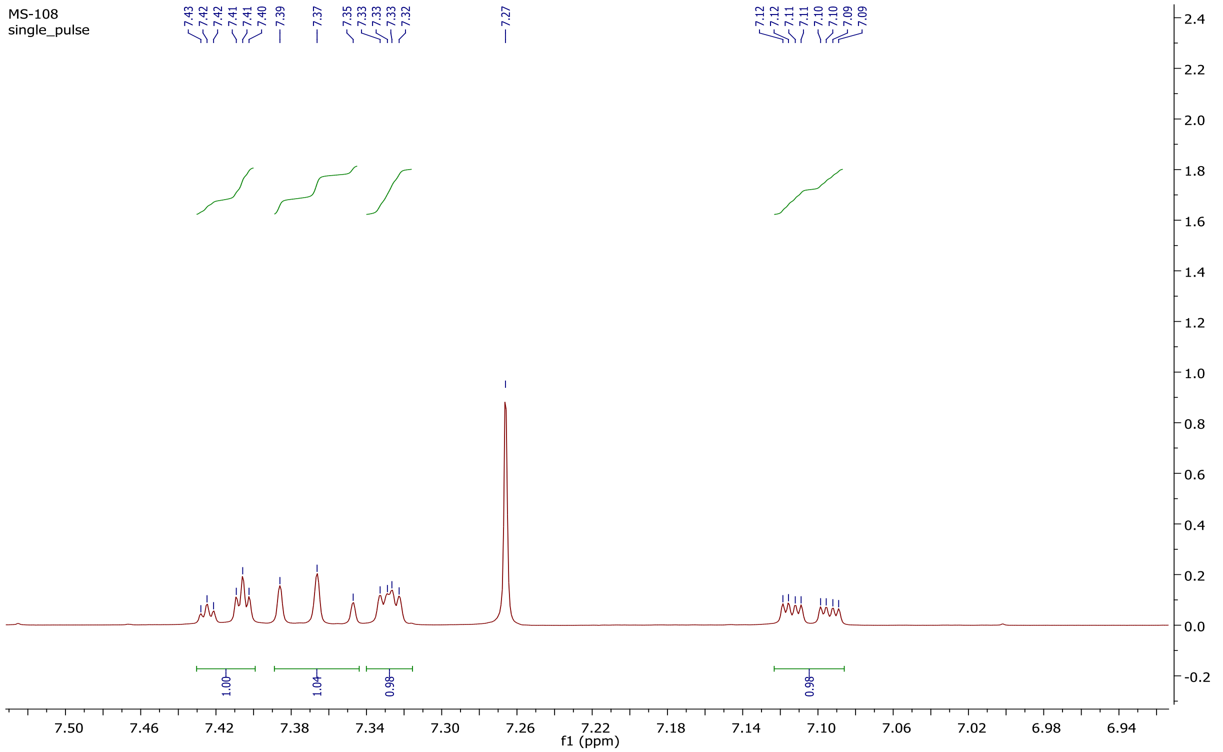

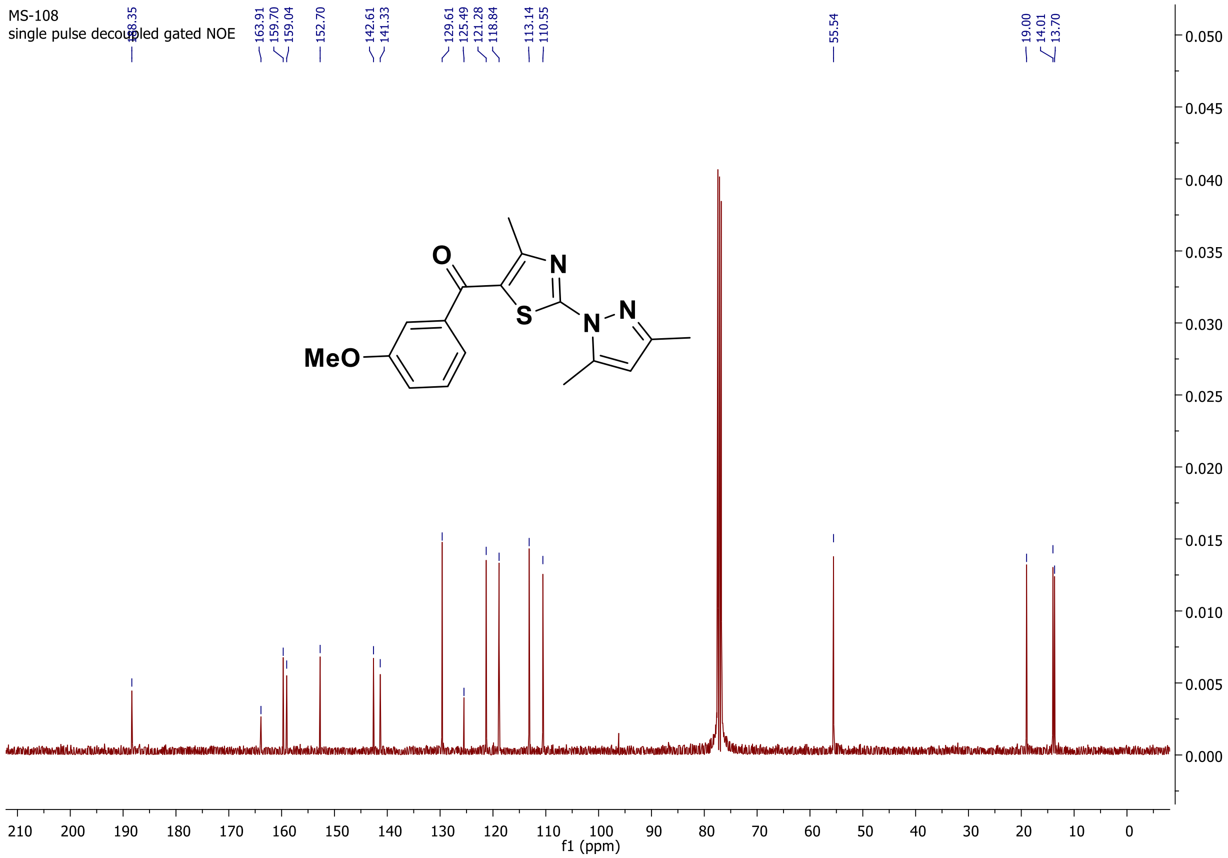
MS-108
single pulse decoupled gated NOE
188.35
163.91
159.70
159.04
152.70
142.61
141.33
129.61
125.49
121.28
118.84
113.14
110.55
55.54
19.00
14.01
13.70
O
N
S
N
N
MeO
f1 (ppm)

5-(2-Methoxybenzoyl)-4-methyl-2-(3,5-dimethyl-1*H*-pyrazolyl)thiazole (3i)

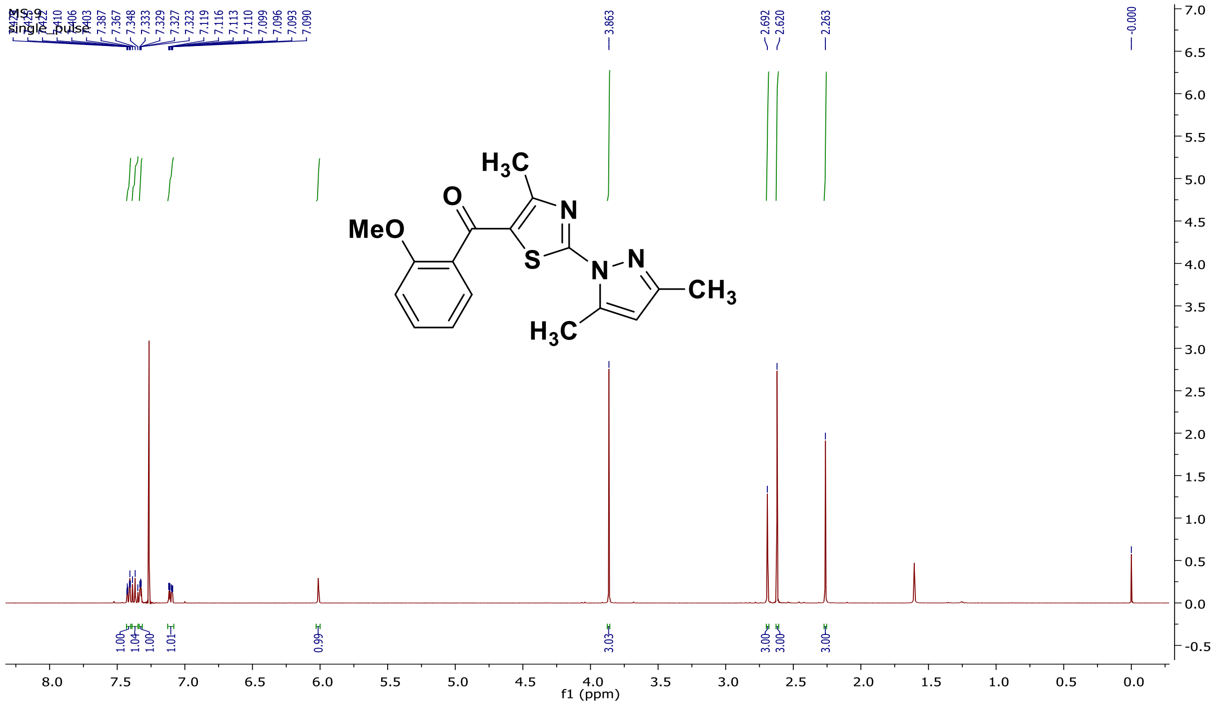

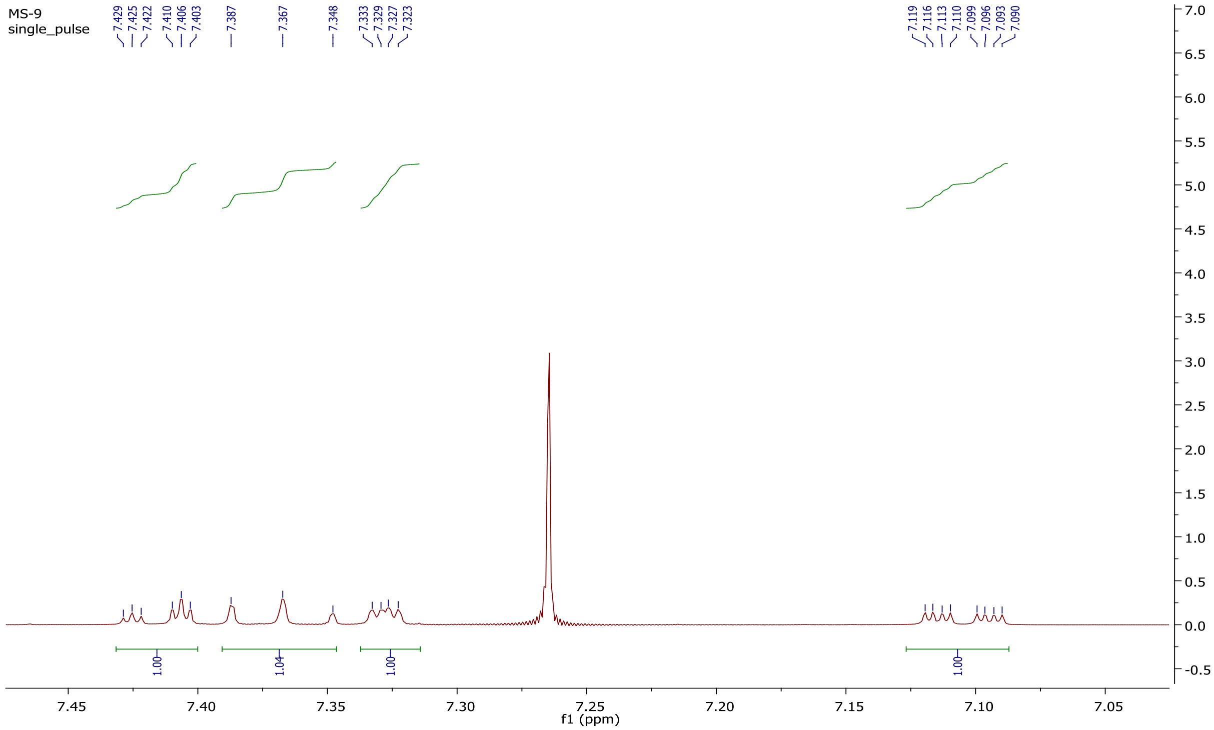

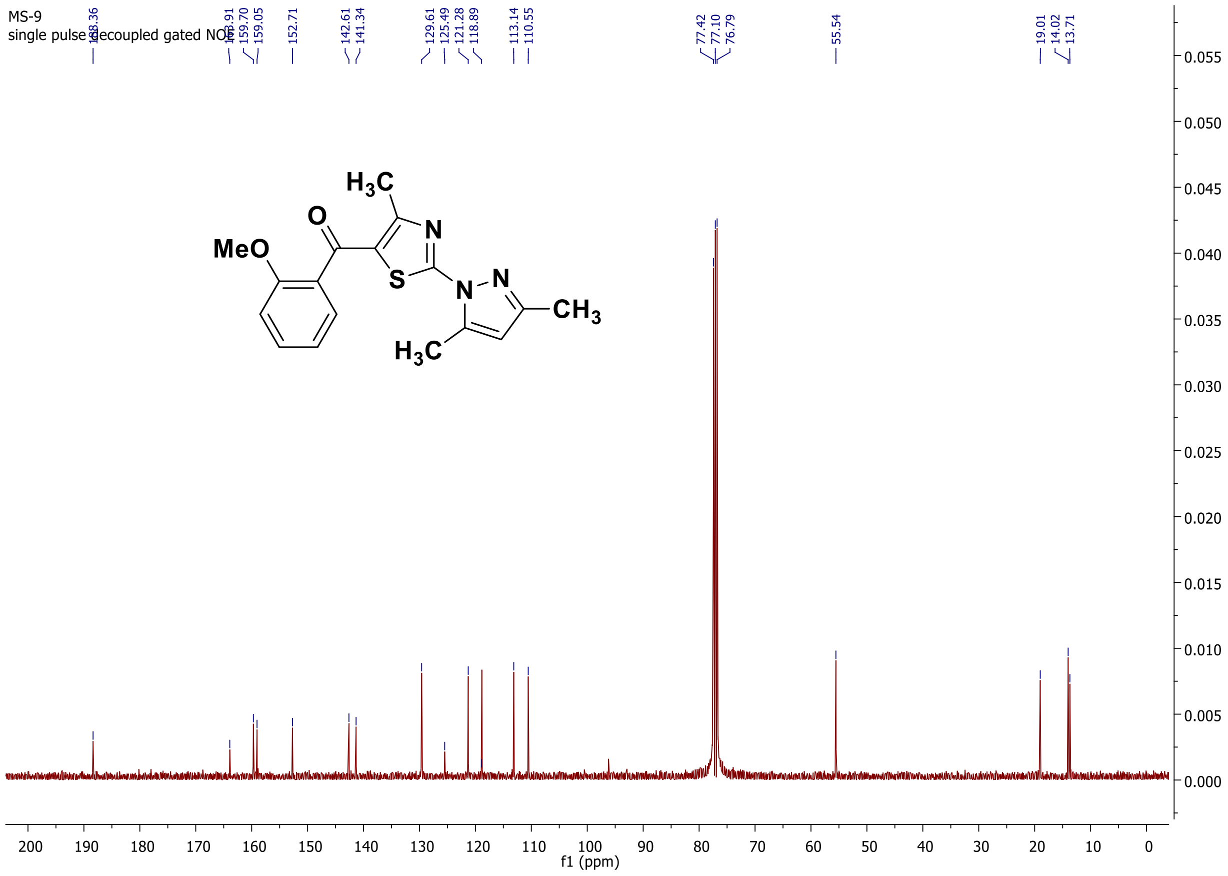
MS-9
single pulse decoupled gated NOE
188.36
163.91
159.70
159.05
152.71
142.61
141.34
129.61
125.49
121.28
118.89
113.14
110.55
77.42
77.10
76.79
55.54
19.01
14.02
13.71
MeO
O
H_3C
N
S
N
N
CH_3
H_3C
0.055
0.050
0.045
0.040
0.035
0.030
0.025
0.020
0.015
0.010
0.005
0.000
200 190 180 170 160 150 140 130 120 110 100 90 80 70 60 50 40 30 20 10 0
f1 (ppm)

5-(2-Thienoyl-4-methyl-2-(3,5-dimethyl-1*H*-pyrazolyl)thiazole (3j)

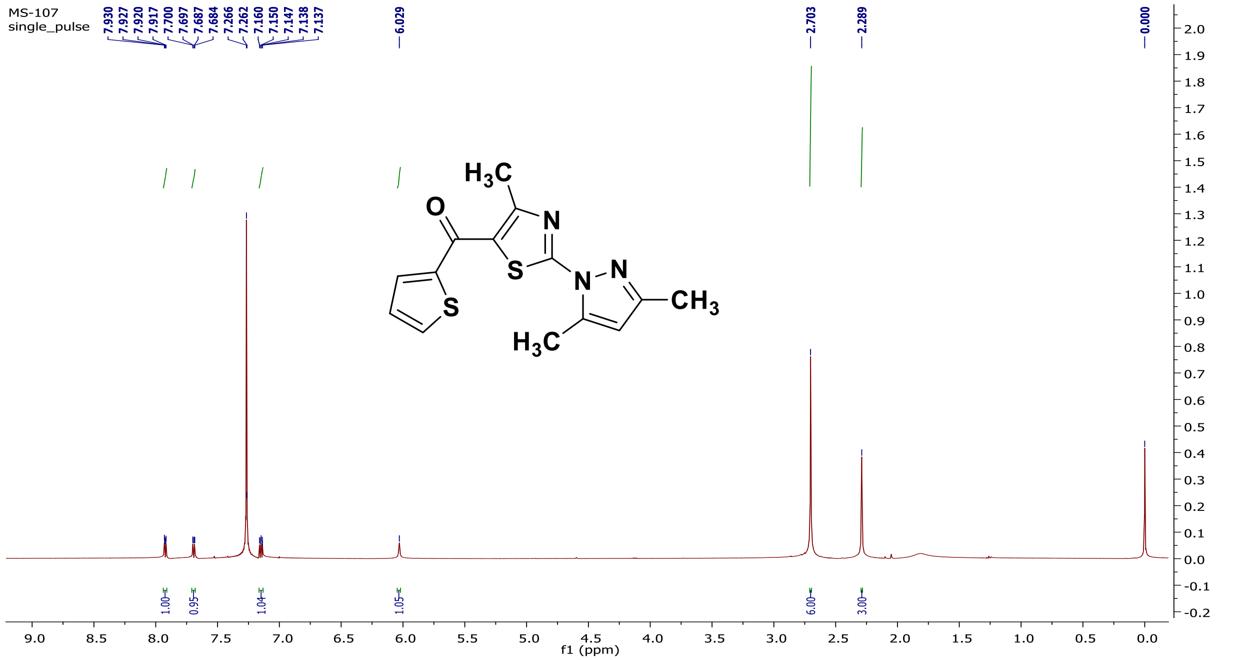

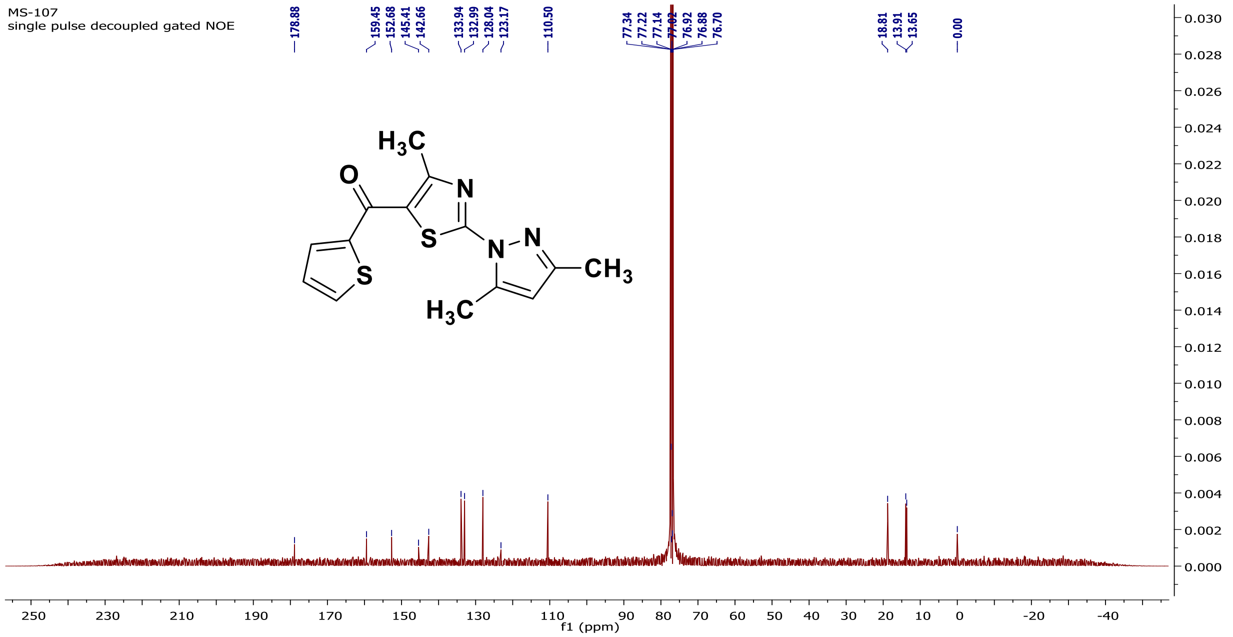

2D NMR (HMBC & HMQC) of 3a

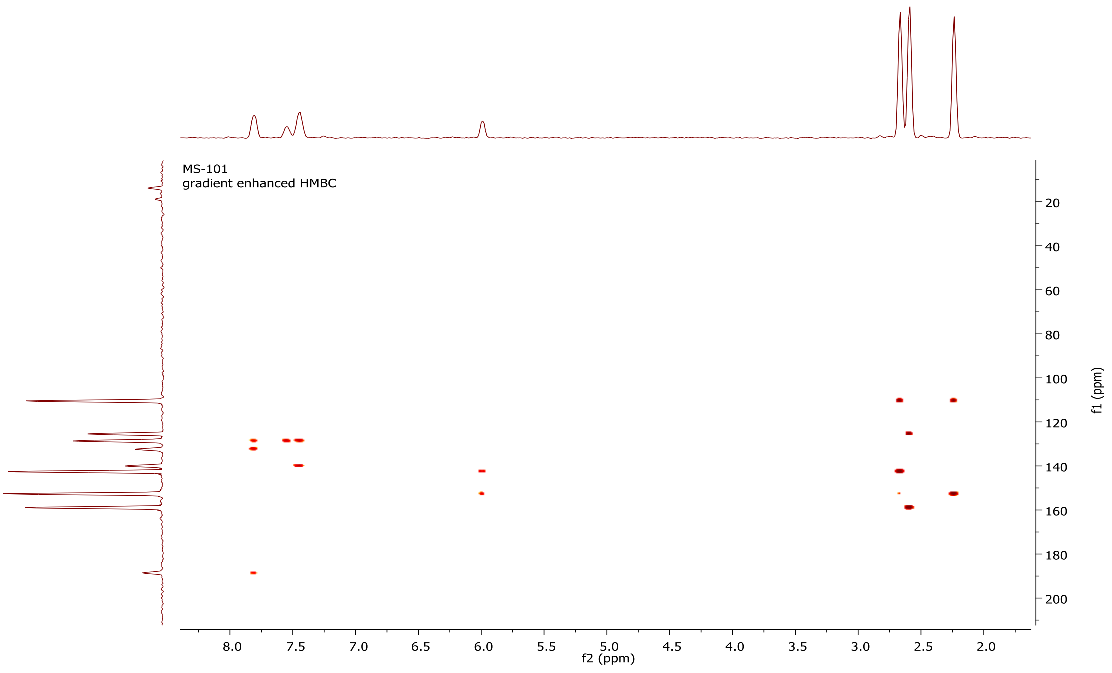

HMBC of **3a**

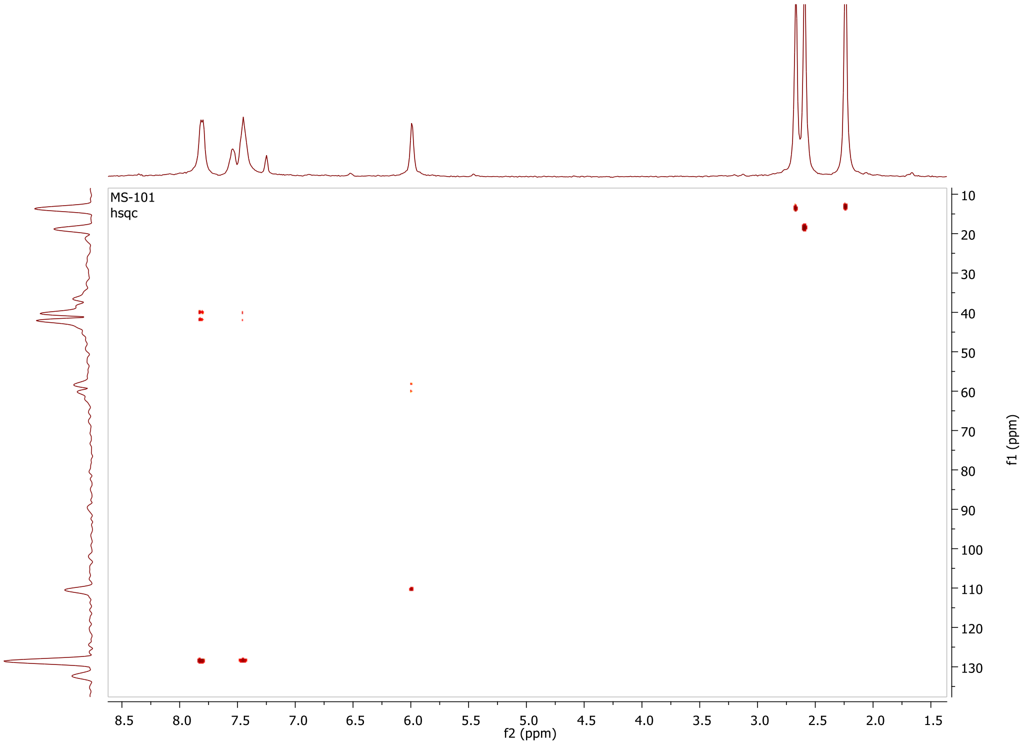

HSQC of **3a**

NMR data in CDCl3, chemical shifts (δ, ppm) for compound 3a (Table S1)

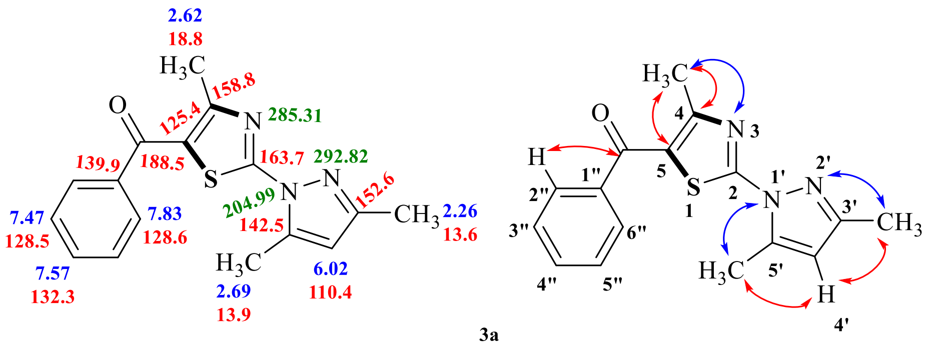

3a

Chemical shifts (δ in ppm)	gs-HMQC correlation	gs-HMBC correlation	Assignments
188.53	---	7.83 (H2”/H6”)<br>2.62(4-Me)	CO
163.74	---		C2
158.88	---	2.62 (4-Me)	C4
152.64	---	6.02(4′H)<br>2.69(5′-Me)<br>2.26(3′-Me)	C3’
142.54	---	6.02(4′H)<br>2.69(5′-Me)<br>2.26(3′-Me)	C5’
139.95		7.47(H3”/H5”)	C1”
132.38	7.57(4”H)	7.83 (H2”/H6”)	C4”
128.64	7.83 (H2”/H6”)	7.57 (H4”)<br>7.83 (H2”/H6”)	C2”/C6”
128.50	7.47 (H3”/H5”)	7.47 (H3”/H5”)	C3”/C5”
125.41		2.62 (4-Me)	C5
110.47	6.02(4′-H)	2.26 (3′-Me)	4′H

		2.69(5′-Me)	
18.89	2.62 (4-Me)	158.88 (C4)	4-Me
		125.41 (C5)	
13.93	2.69 (5′-Me)	6.02(4′-H)	5′-Me
13.63	2.26 (3′-Me)	6.02(4′-H)	3′-Me
204.99	2.69 (5′-Me)		1′-N
292.82	2.26 (3′-Me)		2′-N
285.31	2.62 (4-Me)		3-N

HRMS

5-Benzoyl-4-methyl-2-(3,5-dimethyl-1*H*-pyrazolyl)thiazole (3a)

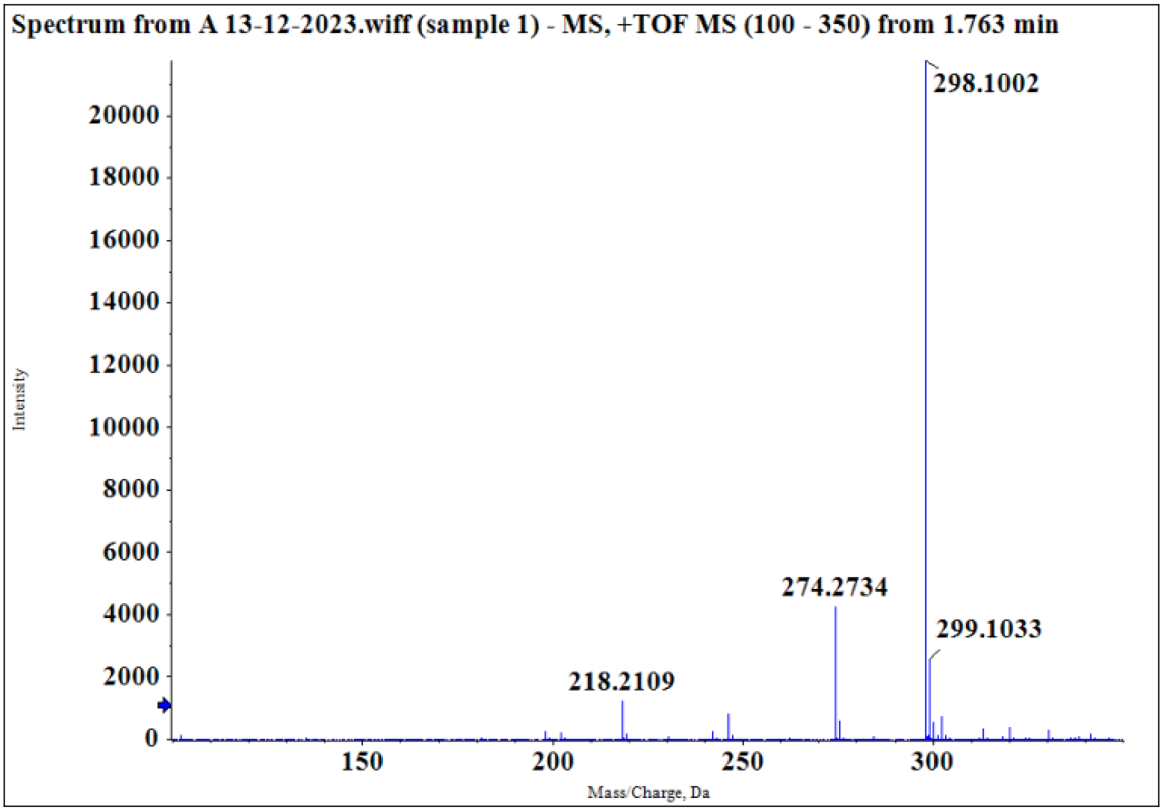

5-(4-Chlorobenzoyl)-4-methyl-2-(3,5-dimethyl-1*H*-pyrazolyl)thiazole (3c)

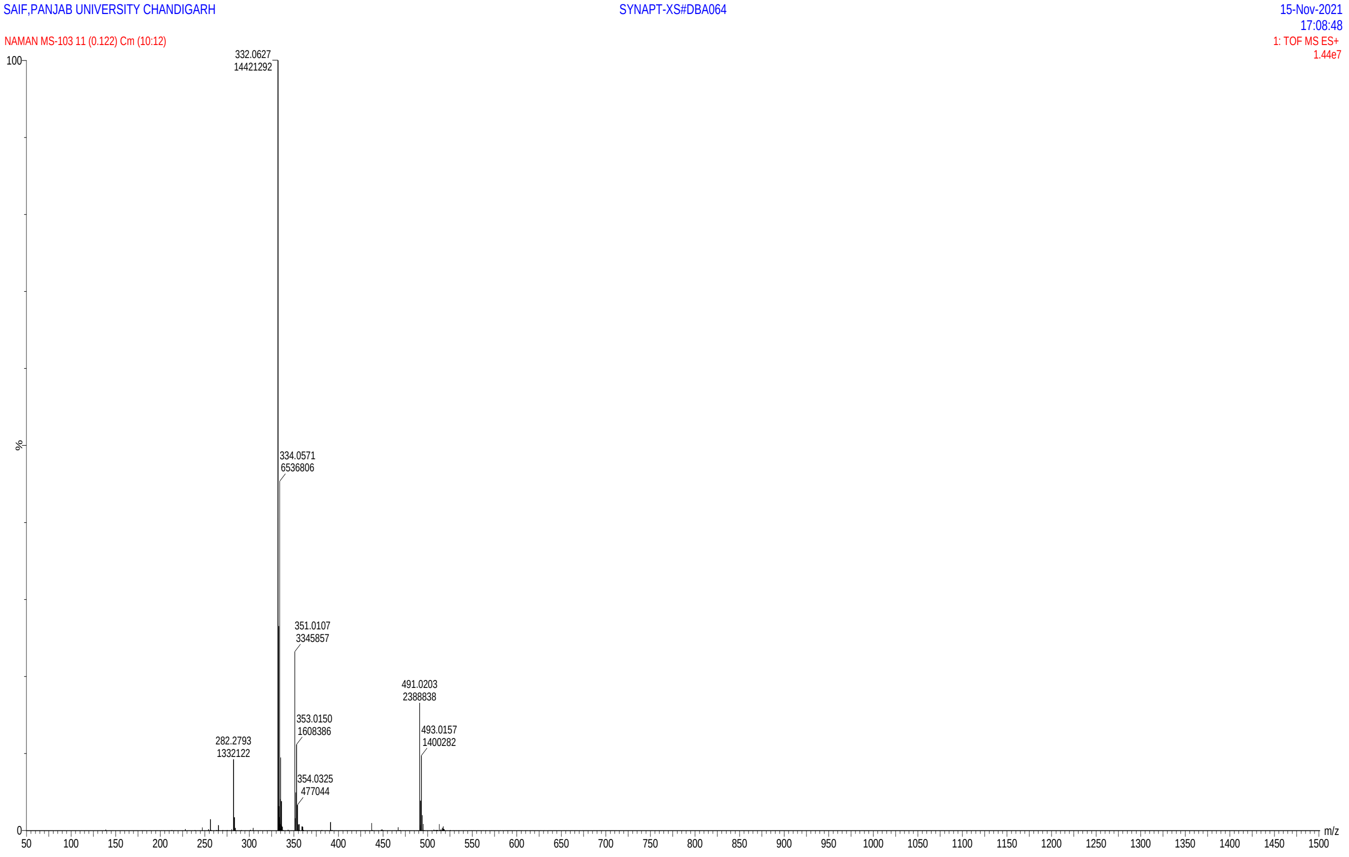

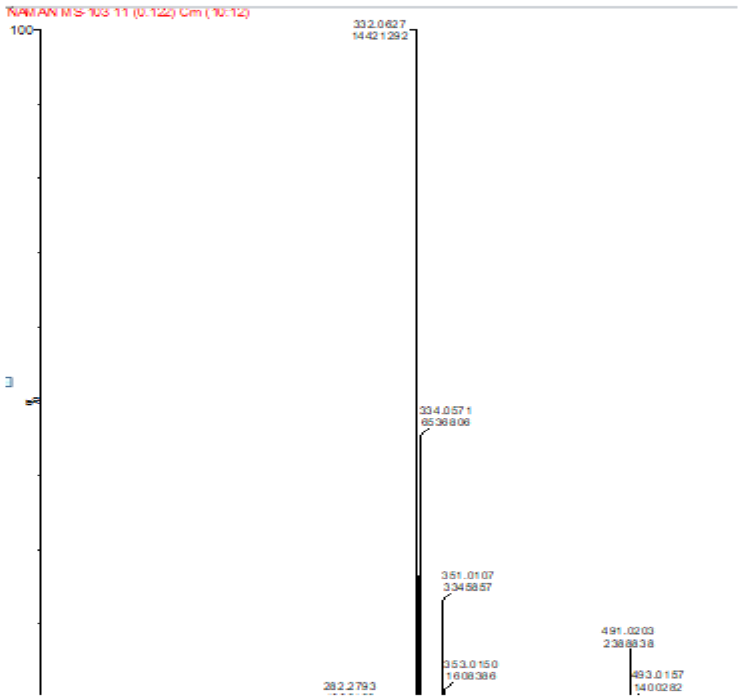

5-(4-Bromobenzoyl)-4-methyl-2-(3,5-dimethyl-1*H*-pyrazolyl)thiazole (3e)

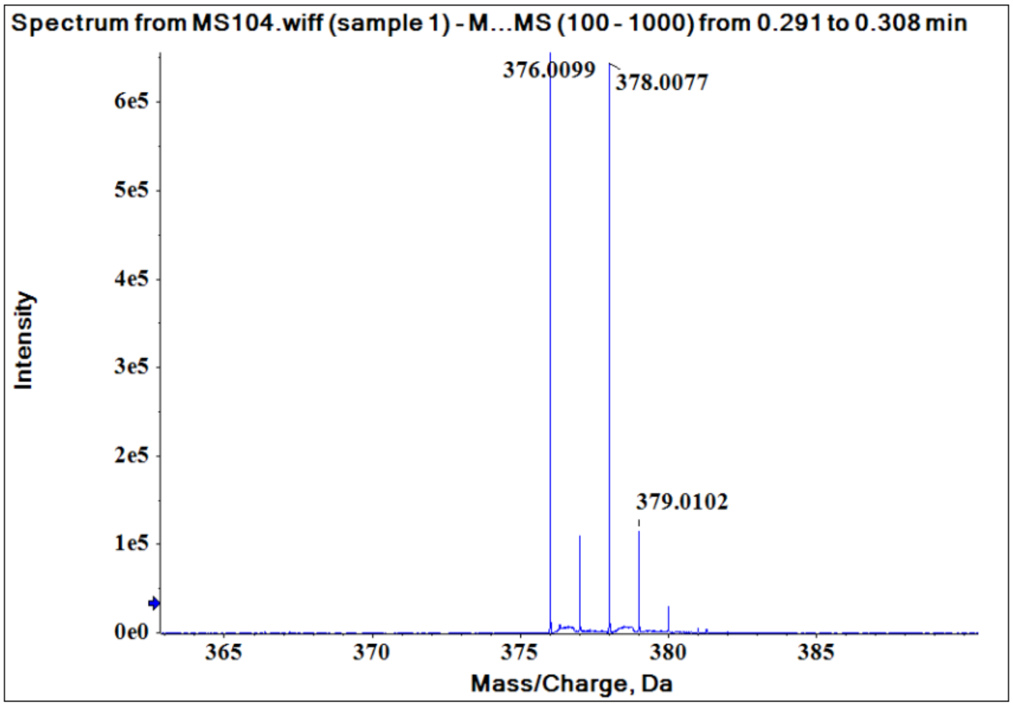

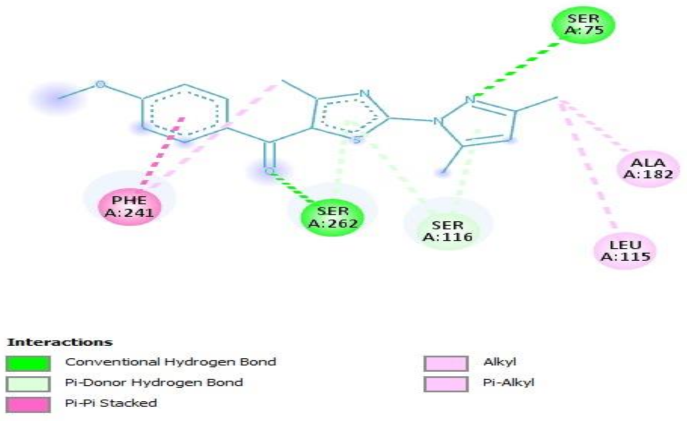

Figure S1: 2D molecular interaction of S. aureus (PDB ID: 3HUN) with **3g**

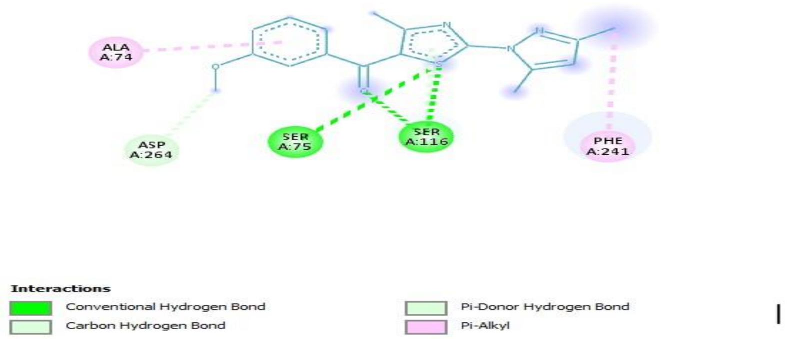

Figure S2: 2D molecular interaction of S. aureus (PDB ID: 3HUN) with **3h**

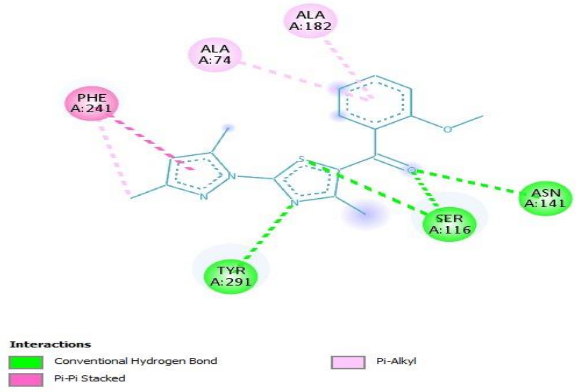

Figure S3: 2D molecular interaction of S. aureus (PDB ID: 3HUN) with **3i**
